# Integrative pan-cancer analysis of AKT1 and structure-based drug design by buckwheat derived compounds

**DOI:** 10.18632/oncotarget.28922

**Published:** 2026-10-12

**Authors:** Ramjan Sheikh, Punam Chowdhury, Ekhlas Uddin, Jarin Tasnim, Mobin Mahmud Shawon, Sohela Faisal, Abu Sayed, M. Salahuddin, Abrar Farhan Fuad

**Affiliations:** ^1^Plasma Cell Research Center, Dhaka 1341, Bangladesh; ^2^Department of Physiology, Biochemistry and Pharmacology, Chattogram Veterinary and Animal Sciences University, Chittagong 4000, Bangladesh; ^3^Department of Biochemistry and Molecular Biology, Gono Bishwabidyalay, Dhaka 1342, Bangladesh; ^4^Department of Applied Chemistry and Chemical Engineering, University of Rajshahi, Rajshahi 6205, Bangladesh; ^5^Department of Pharmacy, East West University, Dhaka 1212, Bangladesh; ^6^Department of Biochemistry and Molecular Biology, Hajee Mohammad Danesh Science and Technology University, Dinajpur 5200, Bangladesh; ^7^Department of Livestock Services, Farmgate, Dhaka 1212, Bangladesh; ^8^Department of Biomedical Engineering, Military Institute of Science and Technology (MIST), Dhaka 1216, Bangladesh

**Keywords:** AKT1, pan-cancer, buckwheat, fagopyrine, molecular docking

## Abstract

AKT1 is a serine-threonine protein-kinase involved in tumour cell proliferation, survival, metabolism, and therapeutic resistance via activation of the PI3K/AKT/mTOR pathway. However, the comprehensive pan-cancer characterization of AKT1 and systematics identifications of natural AKT1 inhibitors remains limited. This study comprehensively evaluated the expression patterns and mutation landscape of AKT1, its promoter methylation, immune infiltration, and clinical significance using the TCGA datasets for 33 cancer types. AKT1 showed significant over-expression in BRCA, HNSC, CHOL, STAD, and UCEC while being under-expressed in PRAD and GBM. Survival analysis demonstrated cancer-specific prognostic associations of AKT1 expression, and cBioPortal profiling characterized E17 as having hotspot missense mutations. Promoter methylation profiling revealed widespread hypomethylation that correlates with increased transcription. AKT1 was widely expressed in normal tissues, and its localization was mainly cytoplasmic, varying according to cell type. At the single cell level, expression was detected in malignant, immune, and stromal compartments. AKT1 expression exhibited tumor-specific associations with tumor mutational burden and microsatellite instability. To identify candidate natural inhibitors, this study computationally screened 52 buckwheat-derived phytochemicals with AKT1. Fagopyrine showed the strongest binding affinity (−15.45 kcal/mol) and remained structurally stable and flexible with AKT1 protein at 100 ns MD simulation. ADMET and DFT analyses indicated favorable electronic stability but predicted moderate gastrointestinal absorption, suggesting that further structural optimization is required. In summary, this study reveals that fagopyrine is a promising computational AKT1 targeting lead, but the direct biochemical and cancer cell-line validation experiments required to confirm its AKT1 inhibitory activity and anticancer efficacy remain to be performed.

## INTRODUCTION

Cancer remains one of the most important causes of death worldwide, with ~20 million new cases and ~9.7 million deaths reported in 2022. Although significant progress has been made in precision oncology, many cancer types still offer challenges in terms of therapeutic resistance, recurrence, and metastasis, underlining the necessity for reliable molecular biomarkers and new therapeutic targets [1, 2]. The phosphatidylinositol-3-kinase/protein kinase B/mammalian target of rapamycin (PI3K/AKT/mTOR) signaling pathway is one of the most frequently dysregulated pathways in cancer. AKT1 is one of its isoforms and a central serine/threonine kinase involved in cell proliferation, apoptosis, metabolism, therapeutic resistance, angiogenesis and migration. Alterations of the AKT1 gene can lead to abnormal activation of this gene and the promotion of various types of tumors. However, a comprehensive multi-omics characterization of AKT1 integrating transcriptional, genomic, epigenomic, clinical, immune, and pharmacogenomic features across diverse cancers remains limited [3–5]. Pan-cancer analysis in pathological systems biology based on large-scale data sets like TCGA is an effective framework to capture shared and tumor-specific molecular signatures of diagnostic, prognostic, and therapeutic interest. Combination of transcriptomic, genomic, epigenomic, and pharmacogenomic data can, therefore, be used to obtain a comprehensive understanding of the biology of AKT1 and its therapeutic relevance from multiple cancer types [1, 6].

Natural products remain a rich source of anticancer therapeutics. Common buckwheat (*Fagopyrum esculentum* Moench) contains diverse bioactive phytochemicals with antioxidant, anti-inflammatory, and antiproliferative properties, making it a promising resource for computational drug discovery [7, 8]. Among these compounds, fagopyrine has structural characteristics that make it suitable for protein binding but, due to its known photo-sensitizing properties, it cannot be used directly in therapeutic treatments, and it has been proposed as a lead scaffold for rational drug design [9, 10]. Combining computer-aided drug design (CADD) techniques such as molecular docking, molecular dynamics (MD) simulation, and density functional theory (DFT) with experimental data has emerged as an efficient strategy for prioritizing compounds by estimating their binding affinity, stability, and electronic properties prior to experimental validation [11, 12]. This study comprehensively analyzed AKT1 in all 33 cancer types in TCGA in terms of genetic alteration, promoter methylation, association with survival, immune infiltration, tumor mutational burden (TMB), microsatellite instability (MSI), pharmacogenomic correlation, protein interaction network, and functional enrichment analysis. Further, this study initially included 97 buckwheat-derived phytochemicals, and after structure curation and quality filtering, 52 compounds were retained for virtual screening, molecular docking, ADMET prediction, molecular dynamics simulations, and DFT analyses to identify potential AKT1-targeting lead compounds. Importantly, the present study does not include direct biochemical assays or cancer cell-line experiments to validate AKT1 inhibition or the anticancer activity of fagopyrine. All the findings are computational, and the identified candidate would need biochemical, cellular, pharmacokinetic, and *in vivo* validation prior to therapeutic use.

## RESULTS

### AKT1 expression patterns in normal tissues and human cancers

Physiological expression pattern of AKT1 was explored using transcriptome expression data available in GTEx across various normal human tissues. As shown in Figure 1A, AKT1 was ubiquitously expressed across nearly all normal tissues, although its expression level varied considerably among different tissue types. Several metabolically active and vascular tissues showed relatively high levels of transcript expression whereas multiple brain regions and whole blood showed relatively low levels of transcript expression. Hence, these results show that AKT1 is widely expressed in various tissues but in a physiological manner, indicating a fundamental role in the maintenance of the different cellular processes. Protein expression analysis based on CPTAC immunohistochemistry images revealed higher AKT1 protein expression in tumor tissues compared with matched normal tissues in several cancer types, including uterine corpus endometrial carcinoma (UCEC), colon adenocarcinoma (COAD), and liver hepatocellular carcinoma (LIHC) (Figure 1B). These findings suggest that AKT1 protein expression is elevated during tumor development in multiple malignancies. Subcellular localization analysis using the Human Protein Atlas demonstrated that AKT1 was predominantly localized in the cytoplasm, with clear distribution around the microtubule network and endoplasmic reticulum (ER) in A-431, U-251MG, and U2OS cell lines (Figure 1C). This localization pattern corresponds to the intracellular signalling activities of AKT1. Overall, these findings suggest the tissue specific expression pattern and AKT1 is often overexpressed at protein level in various human tumor types indicating its possible association with the progression of tumors.

**Figure 1:**
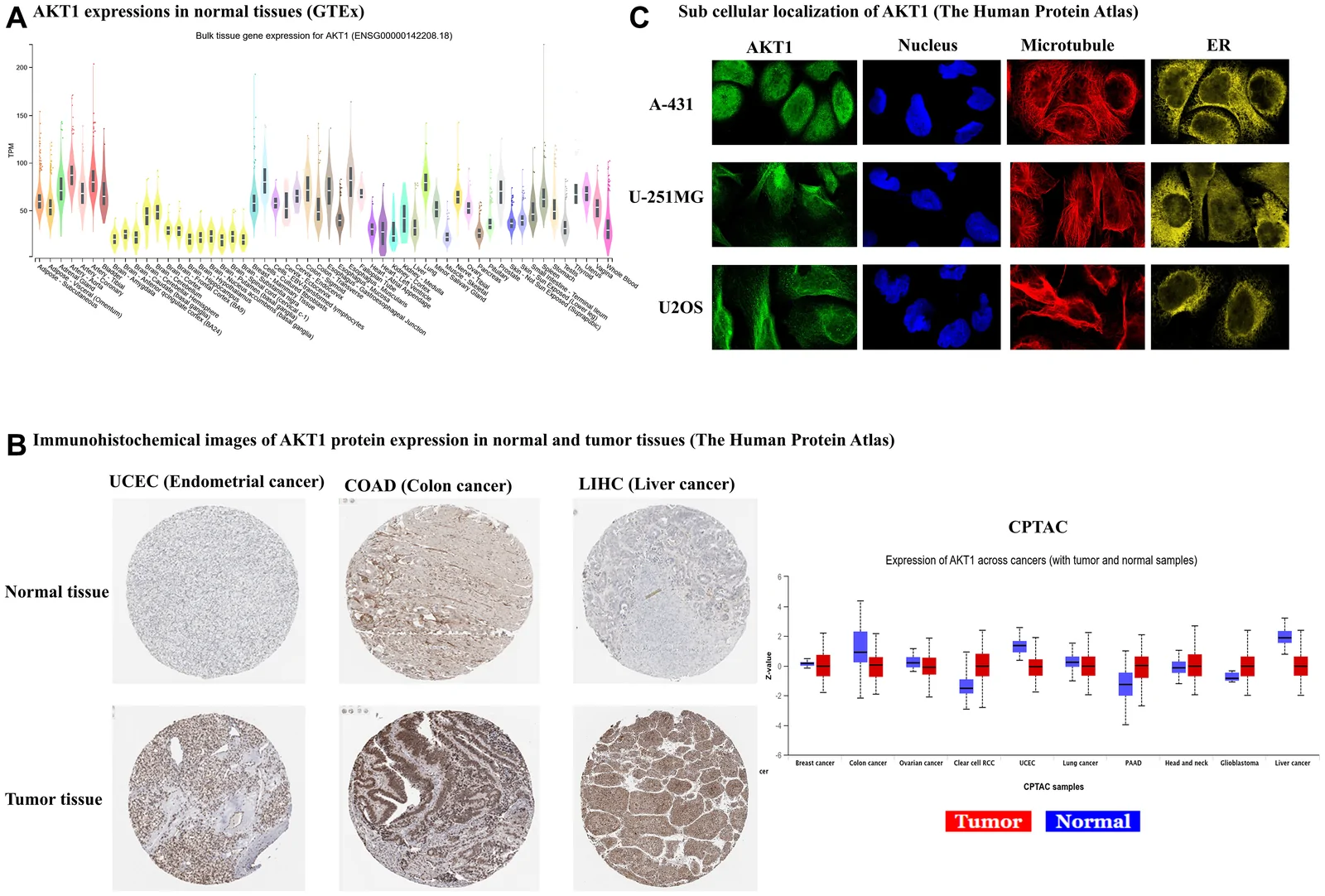
Tissue-specific RNA and protein expression patterns of AKT1 in normal and cancer tissues. (**A**) Heatmap from the GTEx demonstrating tissue-specific RNA expression of AKT1 among normal human tissues. (**B**) Immunohistochemical images of AKT1 protein expression between normal and cancerous tissues, as well as CPTAC-based quantitative analysis of the protein expressions in COAD, LIHC, and UCEC samples; results show higher AKT1 expressions in cancerous than normal tissues. (**C**) Subcellular localization of AKT1 in human cancer cell lines. Immunofluorescence images show the localization of AKT1 (green), microtubules (red), and endoplasmic reticulum (ER; yellow) in A-431, U-251MG, and U2OS cells. The immunohistochemical images in panel B and the immunofluorescence images in panel C were obtained from The Human Protein Atlas (HPA).

### Differential expression and pathological stage-specific analysis of AKT1 in human cancers

To investigate the dysregulation of AKT1 in human malignancies, pan-cancer differential expression analyses were performed using TIMER2.0, UALCAN, and GEPIA2 databases. As shown in Figure 2A, TIMER2.0 analysis revealed that there are considerable differences in AKT1 expression between cancer and normal tissues in multiple cancer types. Several cancers showed high expression of AKT1, and a few cancers showed relatively low expression, suggesting a cancer-type-specific expression pattern. The results were further validated by analyzing transcriptomic data from TCGA and GTEx with the UALCAN platform. Comparative analyses identified significant upregulation of AKT1 in several tumor types, including BRCA, COAD, LIHC, LUAD, STAD, UCEC, PAAD, KIRC, PRAD, and SKCM, compared with corresponding normal tissues (Wilcoxon rank-sum test, *P* < 0.05; Figure 2B). Conversely, under-expression was observed in a small number of cancers, suggesting the heterogeneous expression profile of AKT1 in human tumors. To determine whether AKT1 expression changes during tumor progression, pathological stage analysis was performed using GEPIA2. The expression of AKT1 was significantly associated with pathological stage in KIRC, but not in LIHC, LUAD and BRCA (Figure 2C; *P* < 0.05). The results suggest that AKT1 is widely deregulated across many cancer types but that there may be type-specific links between AKT1 and disease progression.

**Figure 2:**
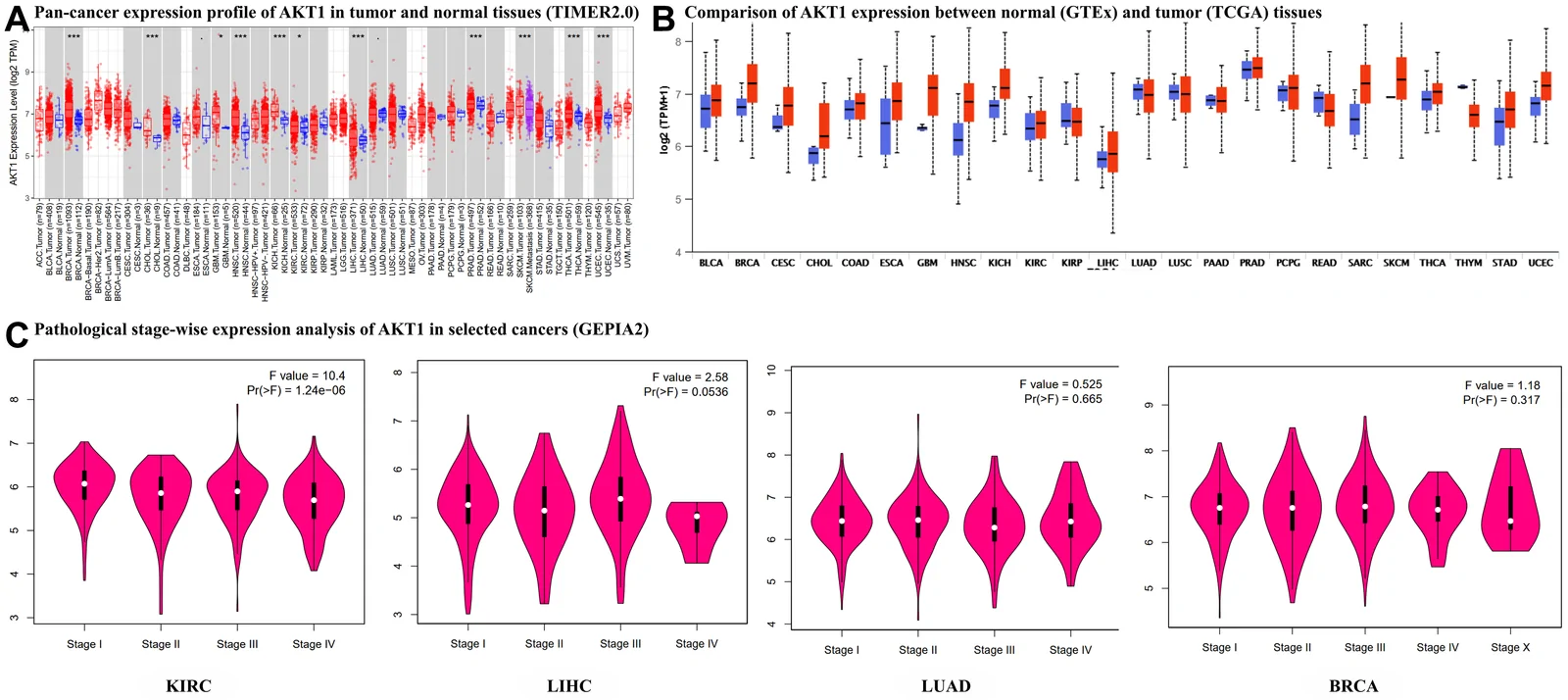
Expression analysis of AKT1 in different tumors and pathological stages. (**A**) Pan-cancer view of AKT1 between normal tissues and tumors via TIMER 2.0. (**B**) Pan-cancer view of AKT1 across all TCGA tumor types analyzed using UALCAN. (**C**) AKT1 expression levels compared with different cancer stages, including KIRC, LIHC, LUAD, and BRCA. ^***^Statistical significance was determined using the Wilcoxon rank-sum test (^*^P < 0.05, ^**^P < 0.01, ^*^P < 0.001).

### Prognostic significance of AKT1 expression in human cancers

To evaluate the prognostic significance of AKT1, overall survival (OS) and disease-free survival (DFS) analyses were performed using the GEPIA2 platform. Heatmap analysis demonstrated that the prognostic impact of AKT1 varied across different cancer types (Figure 3A, 3C). Elevated AKT1 expression was associated with an unfavourable prognosis in LIHC, LUAD, and BRCA, whereas an opposite trend was observed in KIRC, indicating that the prognostic value of AKT1 is cancer-type dependent. Kaplan-Meier analysis further confirmed these findings (Figure 3B). The prognostic significance was determined using Kaplan–Meier survival analysis with the log-rank test (*P* < 0.05). Increased AKT1 expression was significantly associated with poorer overall survival in LIHC, LUAD, and BRCA (log-rank *P* < 0.05), whereas KIRC exhibited the opposite survival pattern. These observations indicate that biological functions of AKT1 vary by tumor type and might represent distinct tumor progression molecular mechanisms. A similar trend was observed for disease-free survival (DFS) (Figure 3C, 3D). Similarly, elevated AKT1 expressions were significantly associated with shorter disease-free survival in LIHC, LUAD, and BRCA (log-rank *P* < 0.05), whereas KIRC showed the opposite trend. Collectively, these results indicate that AKT1 expression possesses prognostic value across multiple malignancies, although its clinical significance is highly dependent on tumor type.

**Figure 3:**
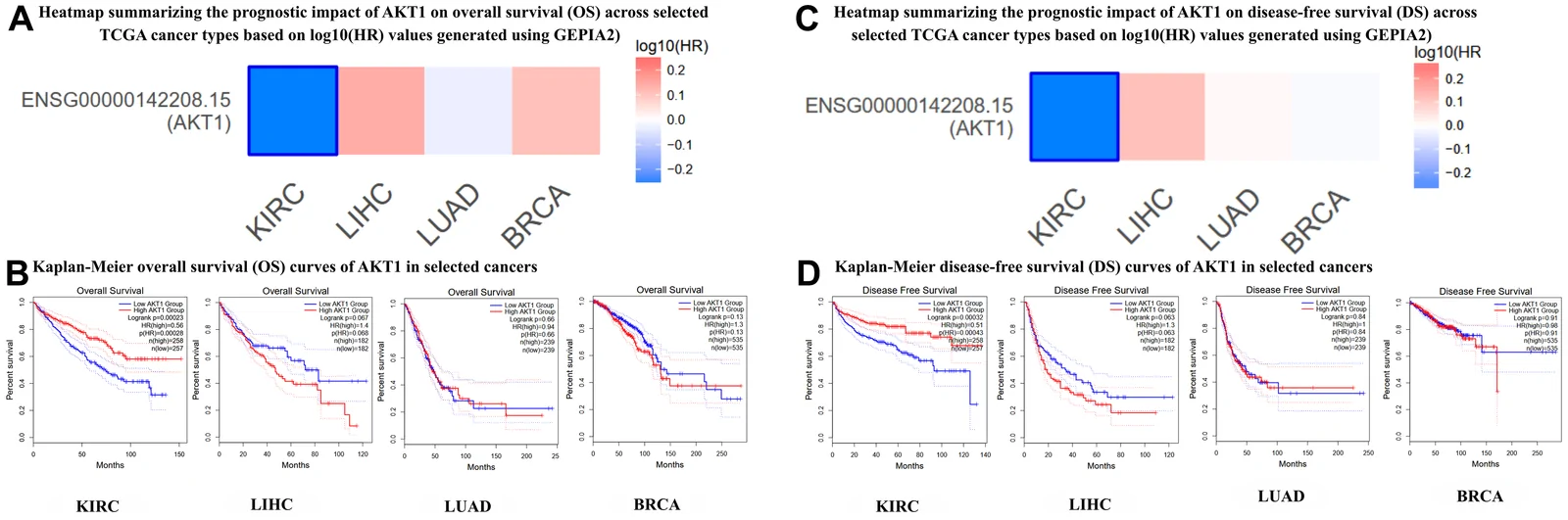
Prognostic significance of AKT1 expression on selected TCGA cancer types. (**A**) Heatmap showing the association between AKT1 expression and overall survival (OS) based on log10(HR) values generated using GEPIA2. (**B**) Kaplan-Meier overall survival curves comparing patients with high and low AKT1 expression in KIRC, LIHC, LUAD, and BRCA. (**C**) Heatmap illustrates the association between AKT1 expression and disease-free survival (DFS) based on log10(HR) values. (**D**) Kaplan-Meier disease-free survival curves for KIRC, LIHC, LUAD, and BRCA. Survival significance was evaluated using the log-rank test.

### Genomic alteration landscape of AKT1 in human cancers

To explore the spectrum of genomic alterations of AKT1 in human malignancies mutation data were evaluated using the cBioPortal platform. As illustrated in Figure 4A, AKT1 presented with alteration frequencies varied across the cancer types of TCGA, with the maximum alteration frequencies observed in UCEC, LUSC, BRCA, CESC, and some other malignancies. Copy number alterations and structural variants were also involved in the genomic alteration profile, with missense mutations being the major class of alteration. To further characterize the distribution of somatic mutations, a lollipop plot was generated using cBioPortal (Figure 4B). Most detected alterations were missense mutations, which were distributed throughout the AKT1 coding sequence, with several recurrent mutation hotspots identified within the pleckstrin homology (PH) and protein kinase domains. These results suggest that mutations preferentially affect functionally important regions of the AKT1 protein. The identified variants were plotted on 3D structure of AKT1 (Figure 4C) to understand the structural location of these mutations. Structural mapping has shown that the recurrent mutations were on the protein surface and in the functionally relevant domains, suggesting that these recurrent mutations may affect the structure and biological function of AKT1. Overall, these observations show that AKT1 is genomically altered in a variety of cancer types, and that missense mutations are the most common mutational event.

**Figure 4:**
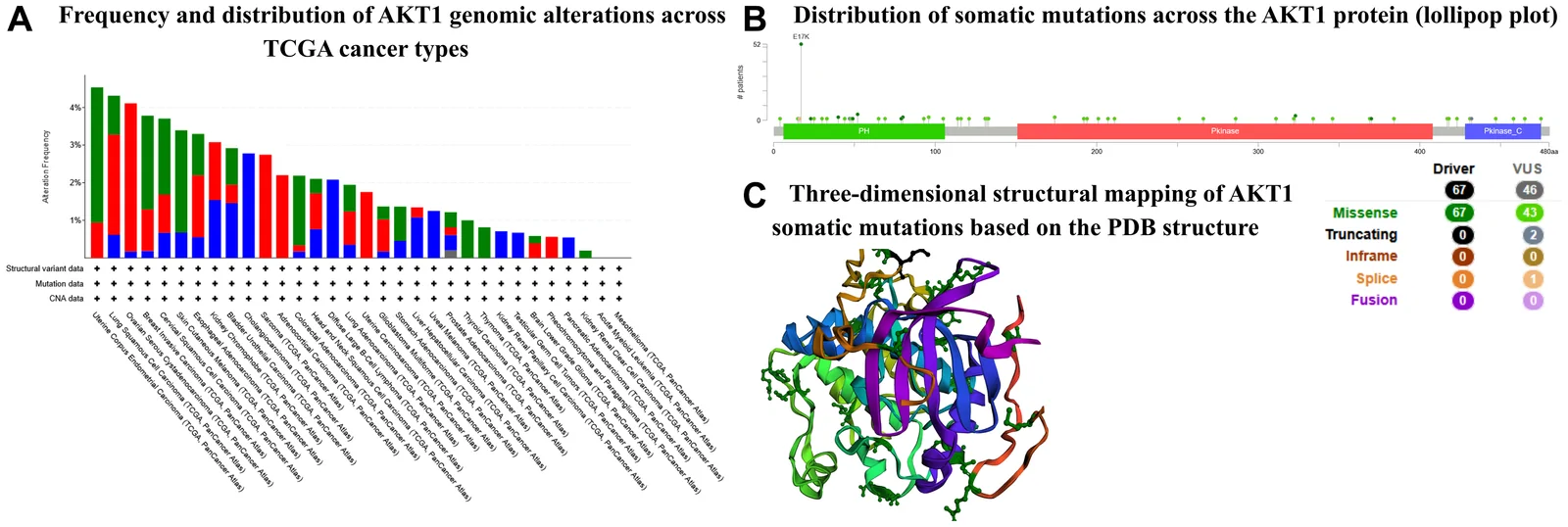
Genomic alteration landscape of AKT1 across TCGA cancer types. (**A**) Frequency and distribution of AKT1 genomic alterations across TCGA cancers generated using cBioPortal. Different colors indicate mutation, copy number alteration, and structural variant frequencies. (**B**) Lollipop plot illustrates the distribution of somatic mutations along the AKT1 protein, highlighting mutations within the pleckstrin homology (PH) and protein kinase domains. (**C**) 3D structural mapping of AKT1 somatic mutations onto the protein structure, showing the spatial distribution of recurrent variants.

### DNA methylation profile of AKT1 across human cancers

To investigate the epigenetic regulation of AKT1, promoter methylation and CpG methylation analyses were performed using UALCAN and methylation datasets across TCGA cancer types. An overview of the genomic and epigenetic changes of AKT1 is provided in Figure 5A, which depicts the distribution of methylation associated changes in different malignancies. Promoter methylation analysis revealed that AKT1 promoter methylation levels were significantly reduced in primary tumors compared with normal tissues in breast invasive carcinoma (BRCA), kidney renal clear cell carcinoma (KIRC), liver hepatocellular carcinoma (LIHC), and lung adenocarcinoma (LUAD) (Figure 5B). These findings indicate that promoter hypomethylation may contribute to the increased expression of AKT1 observed in several tumor types. To further characterize methylation patterns at the CpG site level, individual CpG probes were analyzed in BRCA, KIRC, LIHC, and LUAD (Figure 5C–5F). The methylation levels of the various CpG probes were mostly comparable between normal and tumour tissue, but several CpG probes were found to be significantly differently methylated, indicating epigenetic changes at specific sites in the AKT1 gene. These variations indicate that DNA methylation of AKT1 is heterogeneous across CpG sites and may contribute to cancer-specific transcriptional regulation. Collectively, these findings suggest that altered promoter methylation and CpG-specific methylation patterns may represent important epigenetic mechanisms associated with AKT1 dysregulation in human cancers.

**Figure 5:**
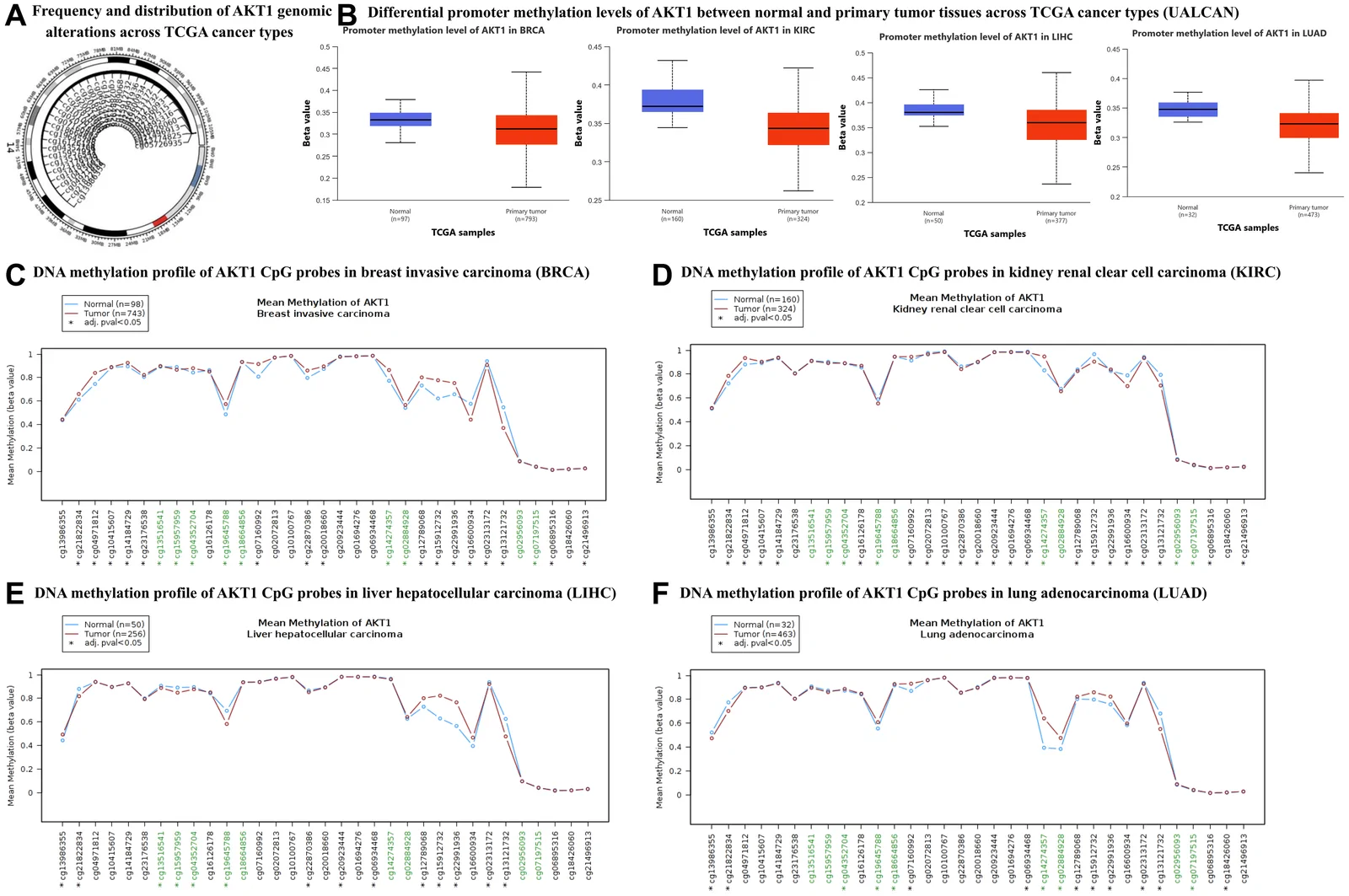
DNA methylation analysis of AKT1 in human cancers. (**A**) Overview of AKT1 genomic and epigenetic alterations across TCGA cancer types. (**B**) Comparison of AKT1 promoter methylation levels between normal and primary tumor tissues in BRCA, KIRC, LIHC, and LUAD using UALCAN. (**C**–**F**) CpG probe-specific DNA methylation profiles of AKT1 in BRCA (C), KIRC (D), LIHC (E), and LUAD (F). Differential methylation levels are shown for individual CpG probes in normal and tumor tissues.

### Association of AKT1 with genomic instability, single-cell expression, and drug sensitivity

To assess the biological and clinical significance of AKT1, its correlation with genomic perturbations, single-cell expression, and drug response were systematically explored across multiple cancer types. Correlation analyses were performed using Spearman’s rank correlation test, and only statistically significant associations (*P* < 0.05) were considered for interpretation. Pan-cancer correlation analysis demonstrated that AKT1 expression was significantly associated (*P* < 0.05) with tumor mutational burden (TMB) and microsatellite instability (MSI) in a cancer-specific manner. Positive correlations with AKT1 were seen with several malignancies with the highest association seen in KICH (R 0.21), SARC (R 0.19), TGCT (R 0.13), CESC (R 0.095), and LUSC (R 0.065) (Figure 6A). Negative correlation was seen in DLBC and READ, showing heterogeneity of AKT1 expression and mutational load between the different tumor types. Similarly, AKT1 expression exhibited variable correlations with MSI. Positive relations were found in PAAD, COAD, LGG, and SARC, while negative relations were found in DLBC, THCA, MESO, and LAML (Figure 6A). Although several associations reached statistical significance, most correlation coefficients were modest, suggesting that the relationship between AKT1 expression and MSI differs among cancer types. Single-cell transcriptomic analysis with the TISCH2 database revealed a widespread expression pattern of AKT1 within the tumor microenvironment (TME) (Figure 6B). Widespread expression of AKT1 was seen in malignant epithelial cells and other stromal and immune cell populations such as fibroblasts, endothelial cells, macrophages, showing moderate to high expression in malignant epithelial cells. To assess the potential therapeutic relevance of AKT1, drug sensitivity analyses were performed using the Genomics of Drug Sensitivity in Cancer (GDSC) and Cancer Therapeutics Response Portal (CTRP) databases. Higher AKT1 expression correlated positively with sensitivity to several therapeutic compounds in the GDSC dataset (Figure 6C). Conversely, CTRP analysis identified several compounds, including BRD-A71883111, CD-1530, ML-1, and timoprazole, that exhibited greater sensitivity in tumors with relatively lower AKT1 expression (Figure 6D). Overall, these results show that AKT1 expression correlates with genomic instability and differential drug response in human cancers, making it a potential cancer stratification marker and therapeutic evaluator.

**Figure 6:**
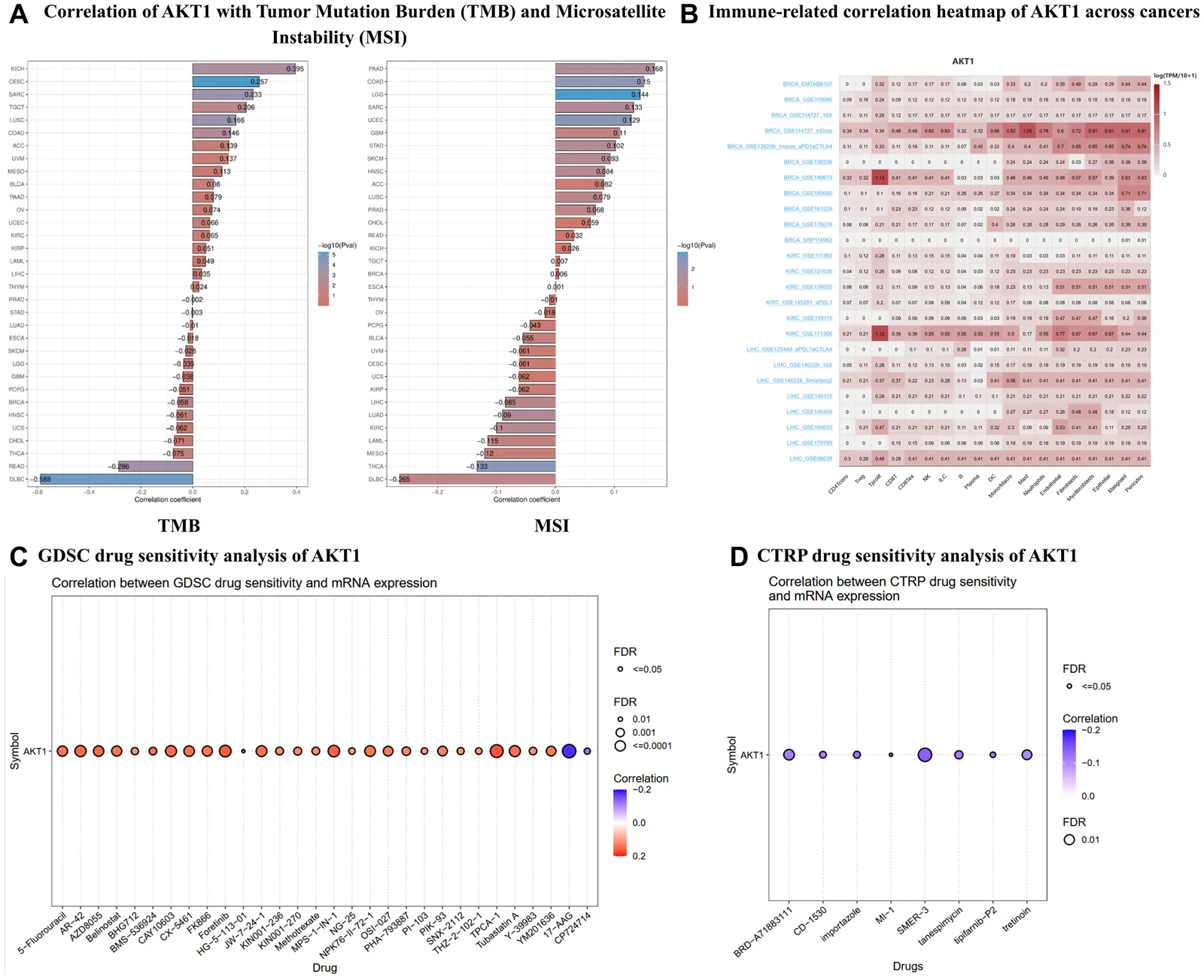
Multi-omics and pharmacogenomic analysis of AKT1. (**A**) Correlation analysis of AKT1 expression with TMB and MSI in pan-cancer. (**B**) AKT1 expression as determined using single-cell RNA-sequencing analysis and its proportion in various cell subtypes of TME. (**C**) Correlation between AKT1 mRNA expression and GDSC drug sensitivity in the GDSC dataset. (**D**) Correlation of AKT1 expression with drug sensitivity in CTRP (Cancer Therapeutics Response Portal).

### Incorporated protein-protein interactions, co-interactions, and enrichment analysis (functions and pathways) of the AKT1 gene

To evaluate the tumorigenesis of AKT1, 50 interacting proteins of AKT1 and 100 associated genes were obtained using BioGrid and GEPIA2, individually. AKT1 gene enrichment analysis was performed based on the combined results. The protein-protein interactions network is depicted in Figure 7A. Two genes show overlap between the two datasets (Figure 7B). Furthermore, the top 100 most positively co-expressed genes were extracted, among which six genes (GPR107, STAU1, NFLX, VPS37C, ZC3H7B, and TM9SF4) were selected based on high expression level (log2 TPM+1) for further validation. The AKT1 expression showed strong positive correlation of GPR107, STAU1, NFLX, VPS37C, ZC3H7B, and TM9SF4 genes (Figure 7C). The heatmap analysis results showed positive correlations of these six genes with AKT1 expression across all cancers (Figure 7D).

**Figure 7:**
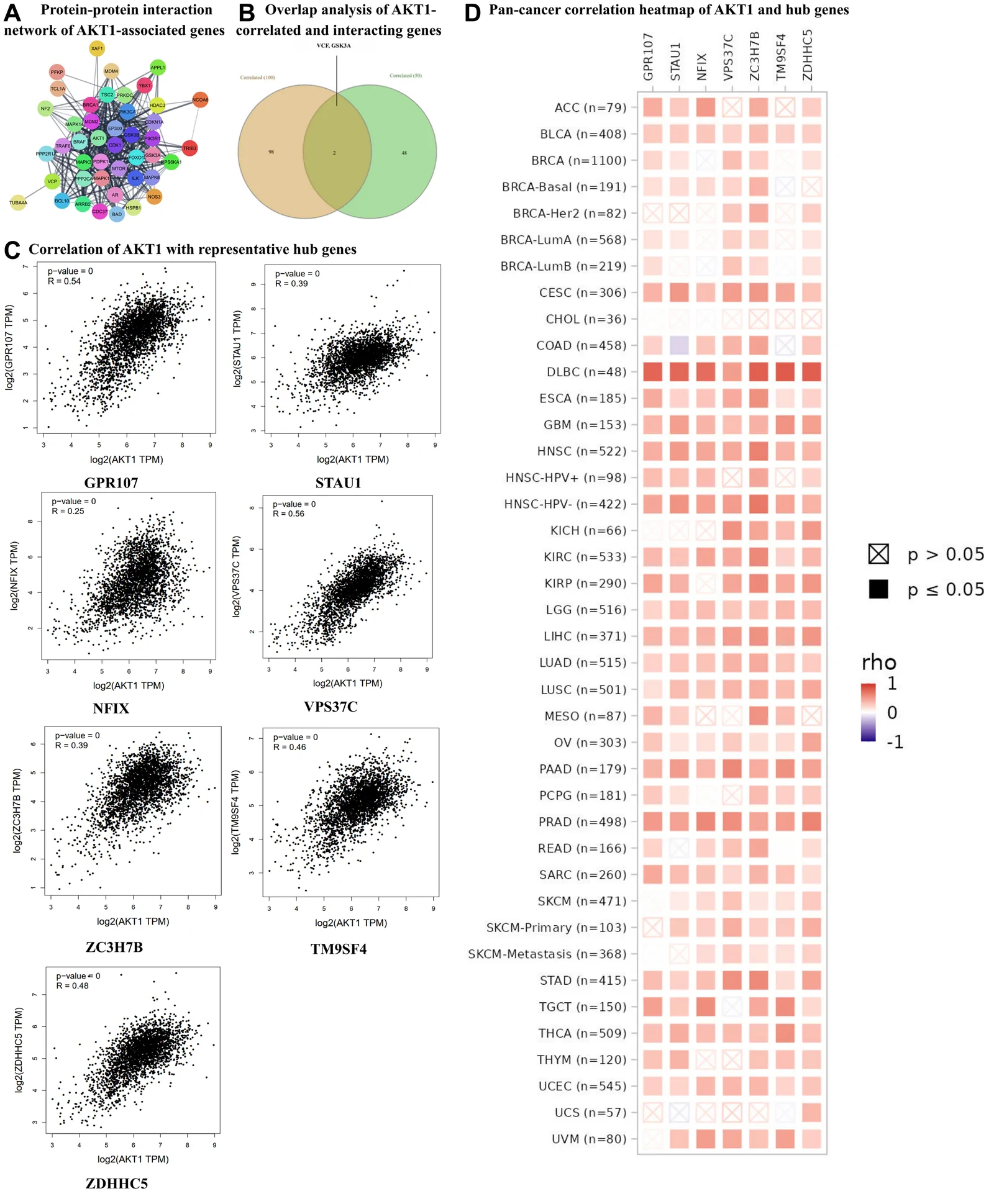
Analysis of co-expressed genes that interacted with AKT1. (**A**) Protein-protein interactions (PPI) of AKT1 identified by BioGrid, (**B**) Overlapped proteins between AKT1-interacted proteins and the co-associated gene sets of the AKT1, (**C**) Correlation analysis of the AKT1 expression with the strongly correlated genes (GPR107, STAU1, NFLX, VPS37C, ZC3H7B, and TM9SF4), (**D**) Heatmap showing the correlation between AKT1 expression with the six co-expressed genes (GPR107, STAU1, NFLX, VPS37C, ZC3H7B, and TM9SF4) in the different cancer types.

Furthermore, AKT1 is associated with various biological processes, including metabolic control, stress response, and immune cell functions (Figure 8A). Particularly, this gene strongly interacted with the negative regulation of the unfolded protein response, modulation of the leukocyte migration, and fatty acid oxidation. AKT1 strongly regulated the human molecular functions, including channel activator activity, phosphatidylinositol-3,4-bisphosphate binding, ATP binding, etc. (Figure 8B). Additionally, AKT1 is associated with different cellular organelles, such as vesicles, microtubule cytoskeleton, cilium, etc. (Figure 8C). AKT1 interacted with various Reactome, Wiki, and KEGG pathways, including PTK6 regulates RTKs and their effectors AKT1 and DOK1, AKT phosphorylates, targets in the nucleus, eNOS activation, caloric restriction and aging, transcription factors regulate miRNAs cardiac hypertrophy, regulation of lipolysis and adipocytes, endometrial cancer, VEGF signaling pathway, etc. (Figure 8D–8F).

**Figure 8:**
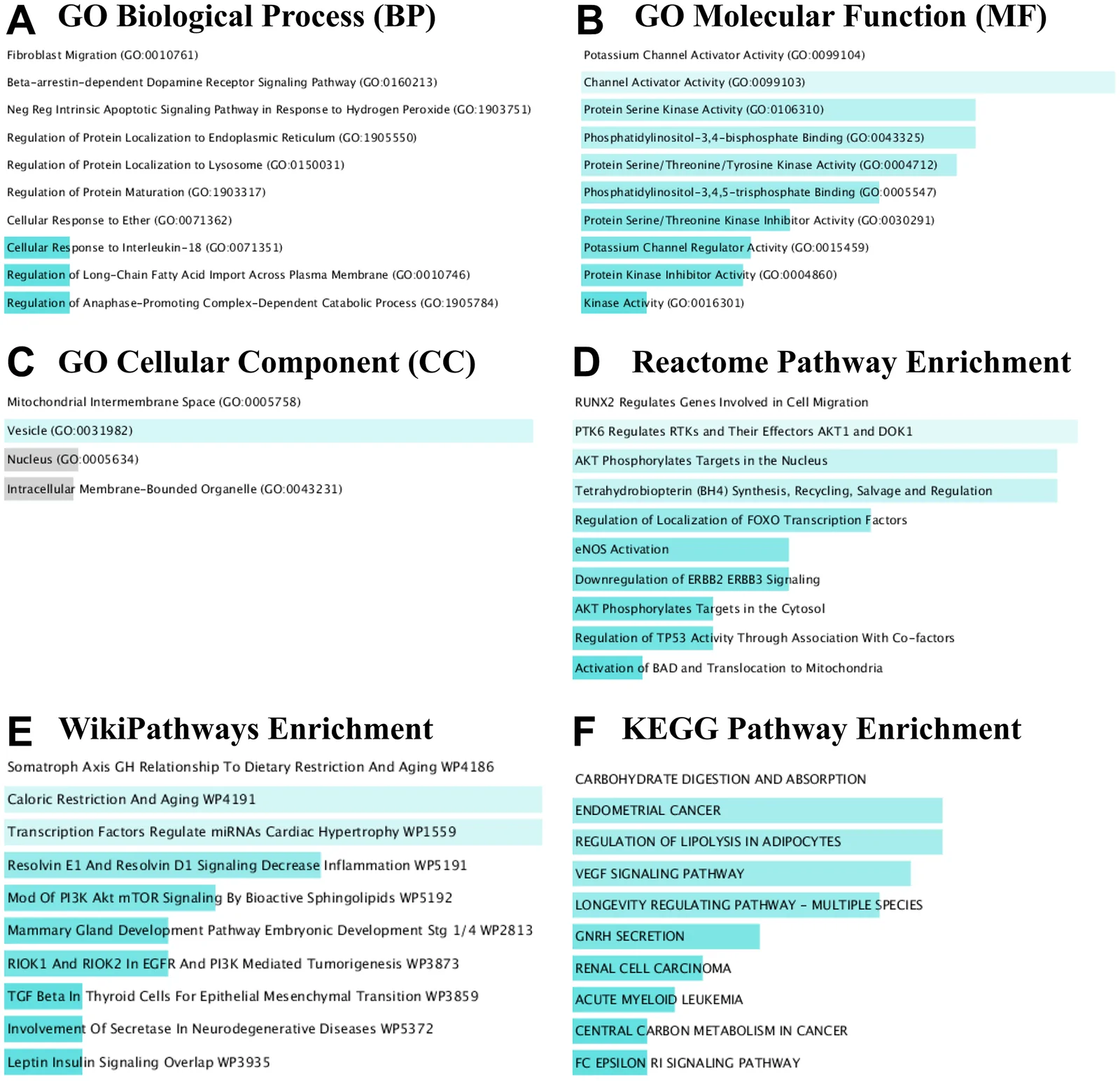
Functional enrichment analysis of AKT1. (**A**) Biological processes. (**B**) Molecular functions. (**C**) Cellular components. (**D**) Reactome pathways. (**E**) Wiki pathways. (**F**) KEGG pathways.

### Molecular docking interaction results of AKT1 with selected buckwheat-derived phytochemicals

This study conducted the molecular docking interactions of AKT1 with buckwheat-derived 52 phytochemicals, and numerous potential candidates with high binding affinities were obtained. Fagopyrine shows the highest binding affinity (-15.45 kcal/mol) and exhibits stable interaction with the active site of AKT1 (Table 1). Further interaction analysis shows that fagopyrine is involved in several key interactions, such as hydrogen bonds and hydrophobic contacts with important amino acids (Asn54, Lys268, Val270, and Leu264) that are required for the catalytic activity of AKT1 (Figure 9). The 3D interaction map elucidated that fagopyrine can fit well into the binding pocket, making extensive Van der Waals contacts and establishing a feasible hydrogen-bond network, which probably enhances its high affinity. These interactions suggest that fagopyrine may represent a potential AKT1-targeting compound and warrant experimental evaluation of its inhibitory activity. Alternately, the native ligand of AKT1 (UC8) interacted with -14.4 kcal/mol binding energy. Another crucial phytochemical, keracyanin, castasterone, α-amyrin, and quercetin also showed good binding affinities and interacting patterns that involved the residues essential for AKT1 activity via hydrogen bonds and hydrophobic contacts (Table 1). However, their binding affinities were comparatively lower, which confirmed the better inhibition efficacy of fagopyrine. The binding affinities and visualization structures of AKT1 with other phytochemicals are provided in Supplementary Tables 1 and 2 and Supplementary Figure 1.

**Table 1:** Summary of molecular docking interactions between AKT1 and the top-ranked buckwheat-derived phytochemicals

Compound ID	Binding affinity (kcal/mol)	No. of H-bonds	Key hydrogen-bonding residues	Major hydrophobic residues	Other important interactions
**UC8**	−14.40	4	CYS310, TYR315, TYR272, CYS296	VAL270, TYR18, TRP80, LEU210, LEU264	π-Cation (ARG273), π-Sulfur (CYS310)
**IMPHY006129 (Fagopyrine)**	−15.45	**3**	VAL271, LEU78, ASN53	LEU264, VAL270, TRP80, GLN79, LYS268, LEU210	π-Donor interaction
**IMPHY000945 (Keracyanin)**	−11.94	5	ASN53, ASP292, ILE290, THR211, TRP80	VAL270, LYS268, LEU210, LEU264	
**IMPHY010558 (Castasterone)**	−11.87	4	ASN54, ASN204, TYR272, THR211	VAL270, ILE84	
**IMPHY011619 (α-Amyrin)**	−11.62	0		VAL270, TRP80	
**IMPHY015054 (Quercetin)**	−11.21	6	SER205, THR211, THR291, ASP292, LYS268, TYR272	TRP80, VAL270, LEU210, LEU264	

**Figure 9:**
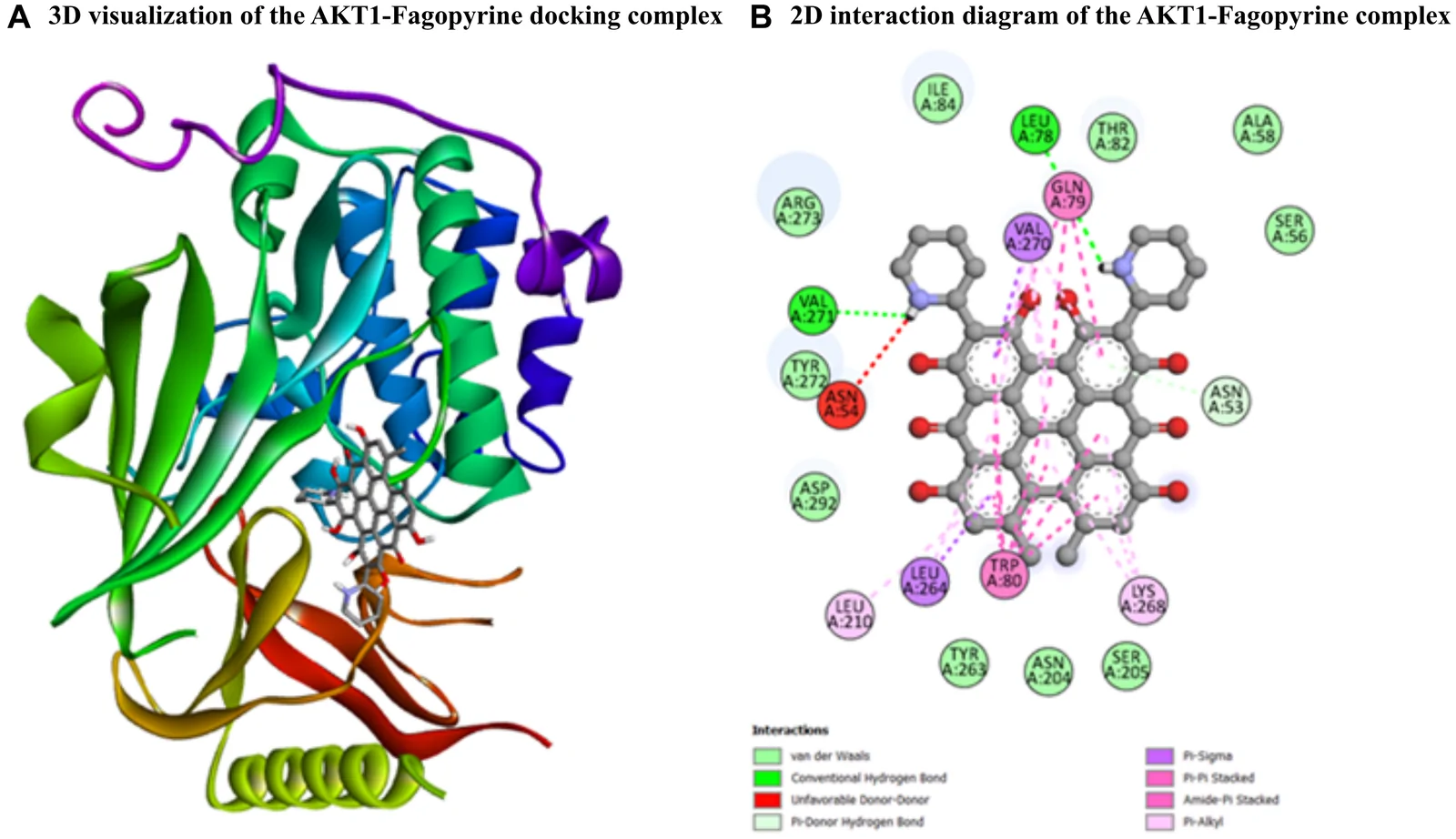
Molecular interaction analysis of AKT1 with fagopyrine. (**A**) Three-dimensional visualization of the AKT1–fagopyrine docking complex showing the binding position of fagopyrine within the active site of AKT1. (**B**) Two-dimensional interaction diagram illustrating the hydrogen bonding, hydrophobic, and other molecular interactions between fagopyrine and the interacting amino acid residues of AKT1.

### ADMET and pharmacokinetics analyses of the fagopyrine

To evaluate the pharmacokinetic and toxicological properties of fagopyrine, ADMET and toxicity analyses were performed using SwissADME and ProTox-III. As summarized in Table 2, fagopyrine exhibited a topological polar surface area (TPSA) of 179.58 Å², two rotatable bonds, and a consensus Log*P* value of 5.47. The compound was predicted to have moderate gastrointestinal absorption and good aqueous solubility. However, lead-likeness assessment identified two violations, including molecular weight (>350 Da) and XLOGP3 (>3.5), indicating that fagopyrine does not fully satisfy the criteria for an ideal lead compound. Additionally, Lipinski’s rule of five analysis revealed that fagopyrine had several violations including molecular weight of 670.71 g/mol, 10 hydrogen bond donors, 8 hydrogen bond acceptors and an X Log *p*-value of 7.39 (Table 3). These results indicate that the compound was not sufficiently drug-like based on the typical measures of drug-likeness in the oral drug discovery process. The toxicity profile predicted by ProTox-III classified fagopyrine as toxicity class IV, with a predicted oral LD_50_ of 330 mg/kg (Table 4). Toxicity endpoint analysis predicted no hepatotoxicity, mutagenicity (Ames), respiratory toxicity, cardiotoxicity, or immunotoxicity, whereas carcinogenicity was predicted to be positive (Table 5). Overall, these results suggest that fagopyrine is reasonably drug-like and has a moderate pharmacokinetic profile, but has several drug-likeness rules violations, along with some predicted toxicity issues, which should be considered for subsequent experimental validation.

**Table 2:** ADMET results of fagopyrine

Ligand	Filters					
	Physicochemical properties		Lipophilicity	Pharmacokinetics	Water solubility	Lead likeness
	TPSA (Å2)	No. of rotatable bonds	Consensus log *p*	GI absorption		
Fagopyrine	179.58	2	5.47	Moderate	Soluble	No; 2 violations: MW>350, XLOGP3>3.5

**Table 3:** Lipinski’s rule of five compliance for selected fagopyrine compound

Ligand Name	Properties				
	Mass, mg/mol	HBD	HBA	XLog P	Molar refractivity
Fagopyrine	670.71	10.0	8.0	7.39	200.50

**Table 4:** Predicted LD_50_ values and toxicity class of the fagopyrine

Compound	LD50 (mg/kg)	Toxicity class
Fagopyrine	330	IV

**Table 5:** In silico toxicity endpoint prediction of fagopyrine

Compound	Toxicity endpoint	Prediction	Tool
Fagopyrine	Hepatotoxicity	No	ProTox-III
	Mutagenicity (Ames)	No	ProTox-III
	Carcinogenicity	Yes	ProTox-III
	Respiratory toxicity	No	ProTox-III
	Cardiotoxicity	No	ProTox-III
	Immunotoxicity	No	ProTox-III

### Molecular dynamics (MD) simulations findings of the AKT1-Fagopyrine complex

To investigate the stability and dynamicity of the system, MD simulation of AKT1 and AKT1-fagopyrine complex was performed for 100 ns. Structural stability and flexibility were evaluated by calculating the root-mean-square deviation (RMSD) and root-mean-square fluctuation (RMSF), respectively. Using thermodynamic properties like overall energy and system density, we assessed the stability and tightness of the complex. Hydrogen bond analysis also revealed that several intermolecular contacts contribute to binding affinity and conformation stability of the AKT1-fagopyrine complex. The combination of these simulations construes an overview of the dynamical and energetic characteristics that characterize the interaction of fagopyrine with the target protein.

The RMSD plot of AKT1 and the AKT1-fagopyeine complex shows the fluctuations of their respective conformations over 100 ns (Figure 10A). The protein by itself has higher RMSDs than 5 Å, indicating that it bends more. The RMSD of 3 Å for the AKT1-fagopyrine complex remains stable, indicating that the formation of the ligand-protein complex is also stabilizing the structure of the protein from fluctuations. The first 10 ns show the initial rise of RMSD, which illustrates the system adjustment, and later the complex is not very fluctuating and stable.

**Figure 10:**
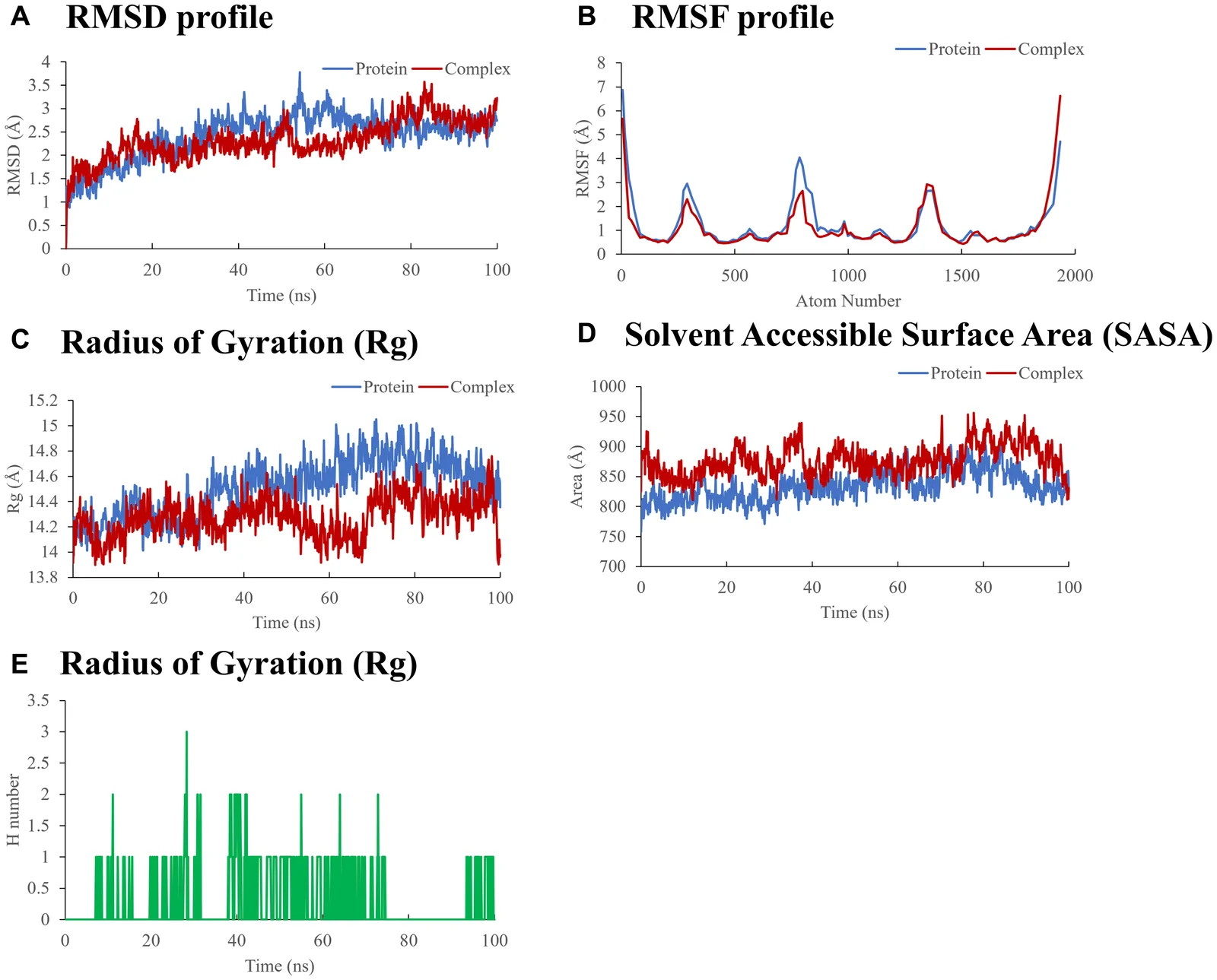
Results from the 100 ns MD simulation of the AKT1-fagopyrine complex. (**A**) Exhibiting the RMSD plot. (**B**) Showing the RMSF plot. (**C**) Showing the radius of gyration plotting. (**D**) Exhibiting the SASA profile. (**E**) Plotting of the hydrogen bond.

The root mean square fluctuation plot (RMSF) depicts the flexibility of the residue over the 100 ns simulation (Figure 10B). Here, the protein (AKT1) has intense motion and is more flexible, notably in the N-terminal region, there are flexible segments. RMSF values of the AKT1-fagopyrine complex display lower values than those of the unbound structure, suggesting stabilization of the protein through reduced residue motion due to ligand binding. This shows the rising trend of ligand (AKT1) binding to the protein in relation to the stability of the protein.

Additionally, the radius of gyration (Rg) profiles of both unbound protein and the AKT1-fagopyrine complex were consistent with little deviation within each condition indicating that the same compact and stable conformation was maintained during the simulation period (Figure 10C). Notably, the complex showed only a slightly lower Rg compared to the protein, which is in line with a less compact and more rigid arrangement of the protein structure upon ligand binding.

The solvent accessible surface area (SASA) was analyzed to evaluate changes in the solvent exposure of AKT1 upon fagopyrine binding (Figure 10D). Throughout the 100 ns molecular dynamics simulation, both the apo protein and the AKT1-fagopyrine complex exhibited relatively stable SASA profiles without abrupt fluctuations. The solvent-accessible surface area (SASA) was consistently higher for the complex than for the apo protein, suggesting a slight increase in SASA upon binding of the ligand. There were some minor fluctuations throughout the simulation, but no major expansion or collapse of structure was detected. These results indicate that binding of fagopyrine does not cause significant conformational shift in AKT1 and that the protein-ligand complex is structurally stable throughout the simulation time.

The hydrogen-bond pattern of AKT1-fagopyrine complex is constantly saturated during the 100 ns simulation, suggesting a relatively linear fluctuation of bonds in the 2–5 range, with occasional peaks in terms of 7 (Figure 10E). Such persistent occupancy implies stable and periodic polar contacts between the ligand and the protein. The absence of prolonged periods without hydrogen-bonding indicates continuous contact is maintained by fagopyrine within the binding pocket for most of the trajectory and is consistent with a stable and energetically favorable binding mode throughout.

### Electronic structure evolution of fagopyrine during molecular dynamics simulation

To investigate whether AKT1 binding alters the electronic properties of fagopyrine, density functional theory (DFT) calculations were performed using the optimized ligand structures obtained before docking (pre-docking), immediately after complex formation (0 ns MD), and after completion of the 100 ns molecular dynamics simulation. The calculated quantum chemical descriptors are summarized in Table 6, and the corresponding HOMO, LUMO and molecular electrostatic potential (MEP) maps are shown in Figure 11. This Frontier Molecular Orbital analysis revealed the HOMO and LUMO orbitals to be mostly localized on the conjugated aromatic structure of fagopyrine across the simulation (Figure 11A, 11B). Although slight redistribution of electron density was observed following docking and during the 100 ns simulation, the overall orbital localization pattern remained conserved, indicating that the ligand maintained its electronic integrity after binding to AKT1. These observations were further confirmed by the quantitative DFT analysis (Table 6). The HOMO energy increased gradually from -5.29 eV in the pre-docking structure to -5.20 eV at 0 ns and -5.12 eV after 100 ns, indicating a slight increase in electron-donating ability during the simulation. Likewise, the LUMO energy shifted from -2.77 eV before docking to -2.67 eV at 0 ns and -2.36 eV after 100 ns, suggesting a modest reduction in electron-accepting capability. Consequently, the HOMO-LUMO energy gap increased slightly from 2.52 eV (pre-docking) to 2.53 eV (0 ns) and 2.75 eV (100 ns), indicating enhanced electronic stability of fagopyrine following prolonged interaction with AKT1. During the simulation the global reactivity descriptors also changed slowly. The ionization potential reduced from 5.29 eV to 5.12 eV, and the electron affinity reduced from 2.77 eV to 2.36 eV, suggesting only minor changes in the electron transfer characteristics. Chemical hardness increased from 1.26 eV to 1.38 eV, whereas chemical softness decreased from 0.397 eV^−^¹ to 0.362 eV^−^¹, further supporting the increased stability and reduced chemical reactivity of the ligand after equilibration. Similarly, the chemical potential increased from -4.03 eV to -3.74 eV, accompanied by a decrease in electronegativity from 4.03 eV to 3.74 eV. Furthermore, the electrophilicity index also decreased gradually from 6.46 eV prior to docking to 6.16 eV at 0 ns and 5.07 eV after 100 ns simulation, which reflects a decrease in electrophilic character after the stabilization of their conformations inside AKT1 binding pocket. To support these numerical descriptors, highly conserved electrostatic features of the MEP surfaces across the three structural states were observed (Figure 11C). Negatively charged electrostatic potential remained associated with the oxygen containing functional groups, while positively charged electrostatic potential remained associated with the hydrogen-rich regions. The electrostatic surface has only slight changes after molecular dynamics simulation, indicating local conformational accommodation and only minor electronic changes. Overall, the combined DFT analyses demonstrate that fagopyrine undergoes only minor electronic reorganization after binding to AKT1 while preserving its overall electronic architecture. The increased HOMO-LUMO energy gap, higher chemical hardness, lower softness, and reduced electrophilicity collectively indicate that the ligand adopts a more electronically stable conformation after the 100 ns molecular dynamics simulation, supporting the stability of the AKT1-fagopyrine complex observed during molecular dynamics analysis.

**Table 6:** Comparative DFT descriptors of fagopyrine before docking, after 0 ns, and after 100 ns molecular dynamics simulation

Descriptor	Unit	Pre-docking	0 ns MD	100 ns MD
HOMO Energy	Hartree	-0.19450	−0.19106	−0.18798
LUMO Energy	Hartree	-0.10180	−0.09821	−0.08686
HOMO Energy	eV	−5.29	−5.20	−5.12
LUMO Energy	eV	−2.77	−2.67	−2.36
Energy Gap (ΔE)	eV	2.52	2.53	2.75
Ionization Potential	eV	5.29	5.20	5.12
Electron Affinity	eV	2.77	2.67	2.36
Chemical Hardness (η)	eV	1.26	1.26	1.38
Chemical Softness (S)	eV^−^¹	0.397	0.397	0.362
Chemical Potential (μ)	eV	−4.03	−3.94	−3.74
Electronegativity (χ)	eV	4.03	3.94	3.74
Electrophilicity Index (ω)	eV	6.46	6.16	5.07

**Figure 11:**
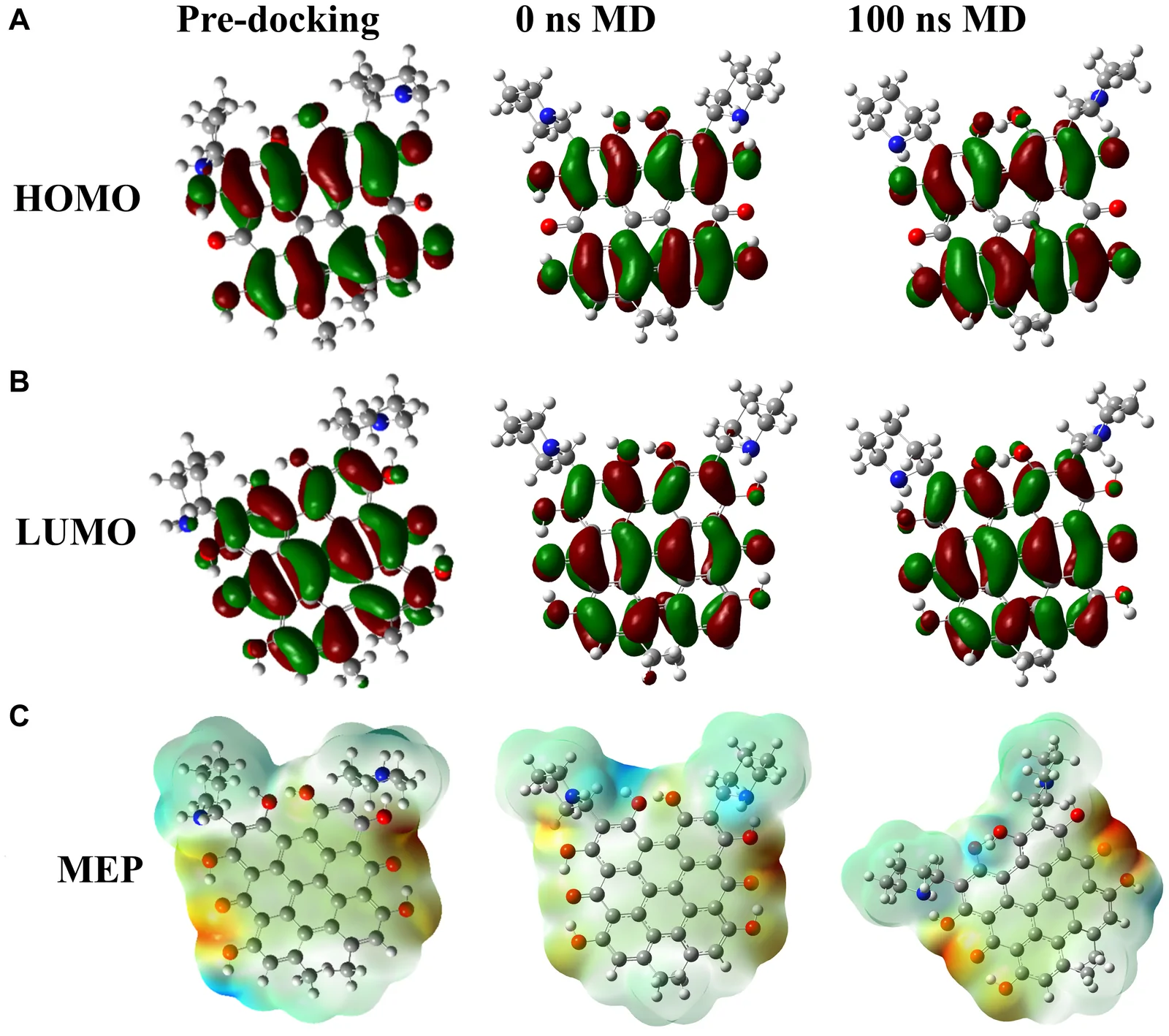
Density functional theory (DFT) analysis of fagopyrine before docking and during molecular dynamics simulation. (**A**) Highest occupied molecular orbital (HOMO) distributions of fagopyrine in the pre-docking, 0 ns molecular dynamics (MD), and 100 ns MD conformations. (**B**) Lowest unoccupied molecular orbital (LUMO) distributions of fagopyrine in the corresponding structural states. (**C**) Molecular electrostatic potential (MEP) surfaces illustrating the electrostatic charge distribution before docking, immediately after complex formation (0 ns MD), and after 100 ns MD simulation.

## DISCUSSION

AKT1 is a key downstream effector of the PI3K/AKT signaling pathway and regulates multiple biological processes, including cell proliferation, survival, metabolism, angiogenesis, and therapeutic resistance. Dysregulation of this pathway is frequently observed across human cancers, highlighting AKT1 as an attractive therapeutic target. In the present study, we performed a comprehensive pan-cancer analysis integrating transcriptomic, proteomic, genomic, epigenetic, immunological, pharmacological, and structural analyses to evaluate the biological and therapeutic significance of AKT1. Furthermore, structure-based virtual screening, molecular dynamics (MD) simulation, and density functional theory (DFT) analyses identified fagopyrine, a buckwheat-derived phytochemical, as a potential AKT1-binding compound.

This study demonstrated that AKT1 was aberrantly expressed in multiple cancer types and was associated with unfavorable prognosis in several malignancies. These observations are consistent with earlier work indicating that constant activation of AKT1 drives tumor development, resistance to apoptosis, re-programming metabolic pathways, and progression to metastasis via continually active PI3K/AKT signaling [5, 13]. Additionally, reduced promoter methylation observed in BRCA, KIRC, LIHC, and LUAD suggests that epigenetic regulation may contribute to AKT1 overexpression. The heterogeneous distribution of CpG methylation also shows that the expression of AKT1 is controlled by complex epigenetic mechanisms in cancer and not simply by CpG methylation at its promoter region [14]. Beyond tumor cells, AKT1 exhibited significant associations with the tumor microenvironment. Immune infiltration and single-cell transcriptomic analyses indicated that AKT1 is broadly expressed in malignant epithelial cells, with expression in fibroblasts, endothelial cells, macrophages, and other stromal cells. These findings align with other studies showing that PI3K/AKT signaling plays a role in immune regulation, stromal activation, angiogenesis, and tumor progression [15, 16]. Similarly, computational cross-sectional associations among AKT1 expression, tumor mutational burden (TMB), and microsatellite instability (MSI) indicate context-dependent relevance to tumor immunobiology and therapeutic stratification which requires further validation in therapeutic cohorts. Further functional enrichment analyses also strengthened the involvement of AKT1 in different oncogenic pathways, such as PI3K-AKT signaling, apoptosis, cell cycle and focal adhesion, highlighting its pivotal role in cancer biology. Furthermore, drug sensitivity analyses using both GDSC and CTRP databases revealed that expression levels of AKT1 were correlated with differential sensitivity to several therapeutic compounds suggesting that AKT1 could be used as a pharmacogenomic marker for personalized treatment strategies.

Among the studied buckwheat-derived phytochemicals, fagopyrine showed the strongest interaction with AKT1. The discovery of potent lead compounds from plant sources (phytochemicals) has been a target of considerable interest due to their structural variety and ability to target multiple cancer-associated biological processes. Many phytochemicals may affect pathways facilitating proliferation and apoptosis of cancer cells, as well as angiogenesis, inflammation, oxidative stress, and metabolic adaptation. Specifically, relevant to the current investigation, several phytochemicals have been described to affect the PI3K/AKT/mTOR signaling axis, the main mediator of the survival and growth of tumor cells. For instance, quercetin was found to be antiproliferative and pro-apoptotic in various experimental models of cancer and was linked to modulation of the PI3K/AKT pathway [17]. The relevance of this mechanism is further supported by evidence from structurally related or chemically relevant phytochemicals included in the present screening framework. Quercetin, which was also identified among the buckwheat-derived candidates in this study and showed a docking score of -11.21 kcal/mol against AKT1, has previously demonstrated anticancer activity through modulation of PI3K/AKT-related signaling. Quercetin has been shown to inhibit cancer-cell proliferation and cancer-cell apoptosis in experimental studies, and to suppress AKT phosphorylation in cancer-cell models [18]. These observations do not establish that quercetin or fagopyrine directly inhibits AKT1 in the present study, but they provide biological context supporting further investigation of phytochemical modulation of AKT-associated cancer signaling. Similarly, keracyanin, which ranked among the stronger docking candidates in the present screening, belongs to the broader anthocyanin-related class of plant pigments. The anticancer properties of anthocyanins, such as by suppressing the proliferation of cancer-cells, inducing apoptosis and interfering with signaling pathways involving PI3K/AKT/mTOR, were extensively studied in experimental models. Although these findings cannot be directly extrapolated to keracyanin or interpreted as evidence of AKT1 inhibition, they provide additional class-level biological rationale for investigating anthocyanin-related buckwheat phytochemicals as potential anticancer leads [19]. The same concept can be applied to α-amyrin, another pentacyclic triterpenoid which had a favorable predicted interaction with AKT1 for the present screening. The chemopreventive and anticancer effects of triterpenoid constituents, particularly α-amyrin, have been explored via the effects on viability, apoptosis, cell cycle control, and signaling pathways of cancer cells. Therefore, the previously reported anticancer potential of chemically related triterpenoid scaffolds may provide context for the prioritization of α-amyrin, and experimental validation would be needed to assess if the predicted interaction of α-amyrin with AKT1 is biologically relevant [20]. Although no previous experimental study has demonstrated direct inhibition of AKT1 by fagopyrine, this computational screening prioritized compounds based on predicted binding affinity and subsequent structural validation rather than prior biological evidence. Nevertheless, evidence from fagopyrin-related preparations provides preliminary biological context for the anticancer relevance of this chemical class. Recently, a study showed that a fagopyrin-containing fraction from *Fagopyrum tataricum* has biologically active photodynamic inactivation activity against the squamous cell carcinoma A431 cells, demonstrating that fagopyrin-containing preparations can have biologically relevant effects in cancer cells [9]. However, such evidence should not be interpreted as evidence of direct AKT1 inhibition by fagopyrine. The present study contributes to the literature and suggests AKT1 as a potential molecular target for further exploration of fagopyrine, as opposed to previously reported direct evidence of target validation. Molecular docking and 100 ns MD simulation consistently demonstrated stable protein-ligand interactions, with RMSD, RMSF, radius of gyration, SASA, and hydrogen bond analyses collectively indicating that fagopyrine remained stably bound within the AKT1 binding pocket throughout the simulation. Comparative DFT analyses performed using the pre-docking, 0 ns, and 100 ns ligand conformations further supported these findings. Only minor variations were observed in HOMO and LUMO energies during the simulation, whereas the HOMO-LUMO energy gap and chemical hardness increased slightly after 100 ns, accompanied by reduced softness and electrophilicity. The molecular electrostatic potential maps were also very similar, reflecting that minimal and possibly limited electronic reorganization occurred on the surface of the molecule in response to ligand binding, without significantly affecting electronic stability. These observations indicate that after equilibration in the AKT1 binding site, fagopyrine is in a more energetically favorable conformation, consistent with the stability noted during molecular dynamics.

Overall, this study highlights that AKT1 is a clinically relevant pan-cancer biomarker with altered gene expression and epigenetic regulation, along with immune characteristics, genomic instability, and therapeutic response in a variety of malignancies. Integrative computational analyses identified a promising lead compound (fagopyrine) exhibits favorable structural and electronic stability and binds to AKT1. Fagopyrine showed strong binding affinity and structural stability with AKT1, but based on its predicted ADMET properties, additional lead optimization steps are required before its therapeutic application. The relatively high molecular weight and elevated lipophilicity, combined with several drug-likeness rule violations, could impede its oral bioavailability and pharmacokinetic profile. This consideration is particularly relevant because fagopyrins have previously been associated with potential phototoxicity, indicating that favorable computational interaction with AKT1 should not be interpreted independently of the compound’s broader safety profile [10]. Structural optimization by scaffold modification, bioisosteric replacement, or analogue development of AKT1 binding activity and/or physicochemical properties should therefore be a major focus for future medicinal chemistry studies. In addition, formulation strategies such as nanoparticle-based delivery systems or prodrug approaches may further enhance bioavailability and reduce potential toxicity, supporting the future development of fagopyrine-derived AKT1 inhibitors. These results offer a computational basis for future development of buckwheat-derived-AKT1 targeted therapeutics, although more medicinal chemistry optimization and experimental validation are still required.

### Limitations and future directions

Despite the comprehensive multi-omics and structure-based computational framework employed in this study, several limitations should be acknowledged. First, the present work is based entirely on publicly available datasets and computational analyses, and no direct biochemical or cancer cell-line experiments were performed to validate the predicted AKT1-fagopyrine interaction or its anticancer effects. Thus, the docking parameters, molecular dynamics stability, MM/PBSA, and DFT derived electronic parameters were given as evidence for prioritization of compounds and not definitive evidence of AKT1 inhibition or anticancer activity. Second, computational ADMET and toxicity predictions can be helpful in early-stage grounds rule estimates but cannot replace the characterization of pharmacokinetic, toxicity or therapeutic-response profiles, which may only be determined through experiment. Future studies should, therefore, evaluate fagopyrine using biochemical AKT1 kinase or binding assays, followed by dose–response and functional experiments in appropriate cancer cell lines to determine effects on cell viability, apoptosis, AKT phosphorylation, and downstream PI3K/AKT/mTOR signaling. Subsequent pharmacokinetic, toxicity, and *in vivo* efficacy studies will be necessary to establish the translational potential of fagopyrine or optimized analogues as AKT1-targeting anticancer candidates.

## MATERIALS AND METHODS

### Identification of cancer associated with AKT1

AKT1 mutation data were acquired from the Cancer Genome Atlas (TCGA) via the NCI GDC data portal (https://cancergenome.nih.gov/) [21]. Coordinated somatic mutation and copy-number variation (CNV) datasets were accessed by querying the AKT1 gene page. Mutation and CNV distribution plots were generated using the portal, and cancer-type-specific alteration frequencies were extracted directly from the plots (Supplementary Figure 2). Averages of these frequencies were used to identify cancers with recurrent AKT1 dysregulation and to choose tumor types for follow-up analyses.

### Tissue-specific RNA expression analysis of AKT1 gene

The GTEx portal was used to analyze the mRNA expression profile of AKT1 across normal human tissues. Gene expression data were obtained as Transcripts Per Million (TPM) values from RNA-sequencing datasets and visualized using a heatmap with hierarchical clustering. The tissue-specific expression of AKT1 (Ensembl ID: ENSG00000142208) was further examined at the protein level using immunohistochemistry (IHC) images retrieved from the Human Protein Atlas (HPA). Differential protein expressions between normal and tumor tissues were evaluated using the CPTAC module of the UALCAN platform. Immunofluorescence images for the A-431, U-251 MG, and U2OS cell lines were obtained directly from the Human Protein Atlas (HPA) [22]. The original HPA images included fluorescence staining for the nucleus (DAPI), AKT1, microtubules, and the endoplasmic reticulum (ER). Subcellular localization of AKT1 was qualitatively assessed based on the original HPA immunofluorescence annotations. No modifications were made to the fluorescence channels, staining patterns, or localization annotations, and the images were presented as provided by the HPA database.

### Transcriptional expression analysis of the AKT1 gene

TIMER 2.0, a free online tool, was used to explore the differential expression of AKT1 between tumor and normal tissues in samples from the TCGA database [23]. Box plots are used to display distributions of gene expression levels. The number of stars (^*^p-value < 0.05; ^**^p-value < 0.01; ^***^p-value < 0.001) indicates the statistical significance determined by the Wilcoxon test. Expression of the gene in tumor samples is denoted by red boxes and normal samples by grey boxes. Statistical significance between tumor and normal tissues was determined using the Wilcoxon rank-sum test as implemented by the TIMER2.0 platform, and a two-sided *p*-value < 0.05 was considered statistically significant. This study further validated the data using the web program GEPIA2 (http://gepia.cancer-pku.cn/index.html). Furthermore, the expressions of AKT1 between different cancer stages of BRCA, HNSC, KICH, THCS, UCEC, CHOL, and STAD cancers were examined via the analysis module “Stage Plot” in GEPIA2.

### Prognostic analysis of the AKT1 gene

The “Survival Map” module of GEPIA2 was used to explore the role of AKT1 in patient prognosis across pan-cancer [24]. The Cancer Genome Atlas (TCGA) transcriptomic and correlate clinical data were used to investigate the relationship between survival of patients and AKT1 expression. We measured the impact of LAG1 and SPL on Overall Survival (OS) as well as Disease Free Survival (DFS) in a subset of cancer types such KICH, DLBC and PRAD. The patients were divided into high- and low-expression groups according to the median level of expression of AKT1, and the cutoff values for high or low expression were 50% of the mean value. The prognostic significance of AKT1 expression was evaluated using Kaplan-Meier survival curves and the log-rank test. Hazard ratios (HRs), 95% confidence intervals (95% CI), and log-rank *p*-values generated by GEPIA2 were used to interpret survival outcomes. A two-sided *p*-value < 0.05 was considered statistically significant.

### Genetic alteration analysis of the AKT1

Genetic alteration analysis of AKT1 was performed by cBioPortal database, an open-source platform for examining multimodal cancer genomics data, provides genetic mutation data of AKT1, including alteration frequency, mutation type, altered site, and 3D structural mapping of somatic mutations. The frequency and types of gene mutations in TCGA tumors were determined using the cancer types of summary module [24].

### The methylation of the AKT1 promoter and the AKT1-related protein expression analysis

DNA methylation analysis of AKT1 was performed using multiple publicly available databases. The overall promoter methylation profile across human cancers was initially investigated using the SMART database [25], and the results were visualized as a pan-cancer circular plot. Differential promoter methylation levels between normal and primary tumor tissues were subsequently evaluated for breast invasive carcinoma (BRCA), kidney renal clear cell carcinoma (KIRC), liver hepatocellular carcinoma (LIHC), and lung adenocarcinoma (LUAD) using the UALCAN platform based on TCGA datasets. To further investigate site-specific DNA methylation patterns, the mean β-values of individual CpG sites associated with AKT1 were obtained from the Wanderer web server and visualized to compare methylation differences between normal and tumor samples [26]. Spearman correlation coefficients (ρ) together with the corresponding *p*-values were used to evaluate correlation strength and statistical significance.

### Multi-omics and pharmacogenomic analysis of AKT1

Tumor mutational burden (TMB) and microsatellite instability (MSI) correlation analysis was carried out to explore the relationship between AKT1 expression and genomic instability in TCGA projects. The association between TMB and MSI scores were performed by ACBI (https://www.aclbi.com) pan-cancer module that relied on TCGA data. We assessed the association of AKT1 with TMB as well as MSI in each cancer type by Spearman’s correlation. Two-tailed *p*-values were determined to evaluate statistical significance, and results were presented as pan-cancer correlation plots. Furthermore, single-cell RNA sequencing data were interrogated by Tumor Immune Single-Cell Hub 2 (TISCH2) database to explore the cellular and microenvironmental pattern of AKT1 expression. Single-cell AKT1 expression in malignant, immune, stromal cell populations within the TME was evaluated based on cell-type-annotated scRNA-seq datasets. We further performed drug sensitivity analyses to determine the association between AKT1 expression and response to therapy. Pharmacogenomic data from the Genomics of Drug Sensitivity in Cancer (GDSC) and the Cancer Therapeutics Response Portal (CTRP) databases were utilized to determine that there was a statistically significant relationship between AKT1 expression levels and drug response (IC_50_ values) using Spearman’s rank correlation analysis.

### Incorporated protein-protein interactions, co-interactions, and enrichment analysis (functions and pathways) of the AKT1 gene

At first, we identified 50 proteins by BioGRID that are associated with AKT1, then generated a protein-protein interaction (PPI) network in Cytoscape to map out their connections. To support these interaction data, we collected 100 co-expressed genes co-expressed with AKT1 from GEPIA2. The top seven genes were investigated according to statistical significance. We next conducted correlation analysis of AKT1 with these seven candidate genes using GEPIA3, by incorporating TCGA tumor, TCGA adjacent normal (pre-tumor), and GTEx tissues as well for robust cross-dataset validation. In order to detect overlapped and unique interactors, a Venn diagram was created by InteractiVenn after comparing BioGRID-identified proteins and the co-expressed gene set obtained from GEPIA2. We also investigated the cancer association of these 7 candidate genes by constructing a correlation heatmap in various TCGA cancers utilizing the TIMER tool. Then, the functional enrichment analysis of the AKT1 gene was performed by the Enrichr database [27]. Enriched pathways and biological processes were presented by the SR plot, a plot that provided the integrated view of the AKT1-oriented network at the functional level.

### Structure-based molecular docking analysis of the AKT1 and buckwheat-derived phytochemicals

A total of 97 buckwheat-derived phytochemicals were initially collected from the IMPPAT and other publicly available phytochemical databases. After structure curation, duplicate removal, and exclusion of molecules without complete 3D structural data or docking files, 52 unique compounds were selected for molecular docking and further computations. The site-specific molecular docking and virtual screening of selected buckwheat-derived compounds against the promising target AKT1 were carried out using AutoDock Vina through a Perl script in the Ubuntu Linux subsystem to identify the optimal ligand-binding conformations and affinities. For docking execution, all required PDBQT and configuration files for the virtual screenings were maintained in a dedicated directory. The grid map of AKT1 (PDB ID: 7NH5) was centered at X: 10.809, Y: -13.071, and Z: -12.843 with X: 32.25, Y: 36, and Z: 44.25 dimensions using AutoDock Tools 1.5.7 and based on the amino acids of active pockets determined by CASTpFold (https://cfold.bme.uic.edu/castpfold/). The compounds were arranged according to their binding affinity, measured in kcal/mol, to determine how strongly they bind to the target’s catalytic site. A further negative score indicates stronger ligand-receptor interactions. Eventually, the top-ranked protein-ligand complexes were visualized using BIOVIA Discovery Studio Visualizer 2021 Client edition and PyMOL 1.7.4.5 to analyze the binding mode.

### MD simulations of the AKT1-Fagopyrine complex

Molecular dynamics (MD) is a computational technique that has played an important role in the study of biological macromolecules by simulating the dynamic changes occurring over time. The study of ligand-receptor binding interactions by means of MD may contribute to the dynamical information on the structure and physical chemistry of a macromolecular system, as well as some detailed views on the energetics of ligand-receptor binding interactions. Molecular dynamics (MD) simulations were performed on the best docked conformer of the AKT1-fagopyrine complex to analyze stability, flexibility, and binding nature. All simulations were performed with Gromacs 2023 using the CHARMM36m force field and TIP3P water model, and the complex box was placed in a cubic simulation box with 0.15M NaCl. After energy minimization, the system was equilibrated for 10 ps at 300 K (NVT) and then for 10 ps at 1 bar (NPT). Further, the respective post-simulation analysis (RMSD, RMSF, Rg, SASA, and hydrogen bond) was done, which confirmed the dynamic stability and constant binding of fagopyrine in the AKT1 cavity.

### ADMET and toxicity profile analysis of fagopyrine

The pharmacokinetic characteristics of fagopyrine were assessed using the SwissADME (https://www.swissadme.ch/) database [28]. The gastrointestinal (GI) absorption, TPSA(Å2), rotatable bonds, consensus Log p, water solubility, lead likeness, and the Lipinski rules of five for drug compounds (Mass (mg/mol), H Donor, H Acceptor, Log p, and Molar Refractivity) were assessed. Toxicity prediction was performed using the ProTox-III web server (https://tox.charite.de/protox3/), which was used to estimate the oral toxicity class, predicted LD_50_ value, hepatotoxicity, carcinogenicity, immunotoxicity, mutagenicity, and cytotoxicity of fagopyrine.

### DFT analysis of the fagopyrine

Density functional theory (DFT) calculations were employed to understand the electronic structure and reactivity of fagopyrine, the buckwheat natural product which had been found with the highest binding affinity for AKT1 in molecular docking studies. All DFT calculations were performed in Gaussian 09W31 using the B3LYP functional with the 6-31G(d) basis set. For a comprehensive understanding of the electronic properties of fagopyrine in different stages during the interaction with AKT1, DFT calculations have been performed on three typical molecular conformations: (i) the isolated ligand before docking, (ii) the ligand conformation after docking, and (iii) the ligand structure extracted from stable binding pose through 100 ns MD simulation. Full geometric optimization was carried out for all stages before electronic properties were calculated. Analysis of the frontier molecular orbitals (FMOs) were used to evaluate the electron transfer and chemical reactivity properties of fagopyrine based on the energies and distributions of highest occupied molecular orbital (HOMO), lowest unoccupied molecular orbital (LUMO). For the study of molecule stability and reactivity, HOMO-LUMO gaps were computed. Furthermore, the electronic total energy of the optimized structures was calculated to estimate energetic changes due to binding and dynamic stabilization in AKT1 active site.

### Statistical analysis

Statistical analyses were performed using the statistical methods implemented in the respective publicly available databases and bioinformatics platforms. Unless otherwise specified, statistical significance was defined as a two-sided *p*-value < 0.05. Where applicable, false discovery rate (FDR)-adjusted *p*-values were considered to account for multiple hypothesis testing.

## CONCLUSIONS

This study combined transcriptomic, proteomic, genomic, epigenetic, immune, pharmacological, and structural analyses to offer a pan-cancer characterization of AKT1. The results showed AKT1 dysregulated expression in various human cancer types, which was correlated with altered prognosis, its promoter methylation status, chromosomal alterations, immune infiltration, genome instability, and differential drug response, indicating its potential as a prognostic marker and therapeutic target. Structure-based virtual screening identified fagopyrine, a buckwheat-derived phytochemical, as the top-ranked AKT1-binding candidate, and subsequent molecular docking, molecular dynamics simulations, MM/PBSA, and comparative DFT analyses consistently supported the structural and electronic stability of the AKT1-fagopyrine complex. However, some of the drug-likeness restrictions that were predicted and the reported phototoxicity of native fagopyrine suggest that it should be regarded as a promising lead scaffold instead of a clinically applicable drug candidate. Future studies should focus on structural optimization and analogue development, followed by biochemical validation and cancer cell-line experiments to determine whether the predicted AKT1 interaction translates into measurable target inhibition and anticancer activity. Subsequent *in vivo* pharmacokinetic, toxicity, and efficacy studies will be required before any consideration of therapeutic translation. Together, these results suggest a computational platform to guide AKT1-targeted drug discovery and forms the basis for further research on investigating buckwheat-derived phytochemicals as anticancer lead compounds. Nevertheless, medicinal chemistry optimization and experimental target validation will be essential before fagopyrine can be considered a clinically relevant AKT1-targeting lead.

## SUPPLEMENTARY MATERIALS


