# Supplementary material for: Integrative pan-cancer analysis of AKT1 and structure-based drug design by buckwheat derived compounds
Source: Oncotarget. 2026 Oct 12;17:437–61. doi: 10.18632/oncotarget.28922 (PMC13718337; doi:10.18632/oncotarget.28922)
Supplement: Supplementary file 1 [file oncotarget-17-28922-s001.pdf]

Integrative pan-cancer analysis of AKT1 and structure-based drug design by buckwheat derived compounds

SUPPLEMENTARY MATERIALS

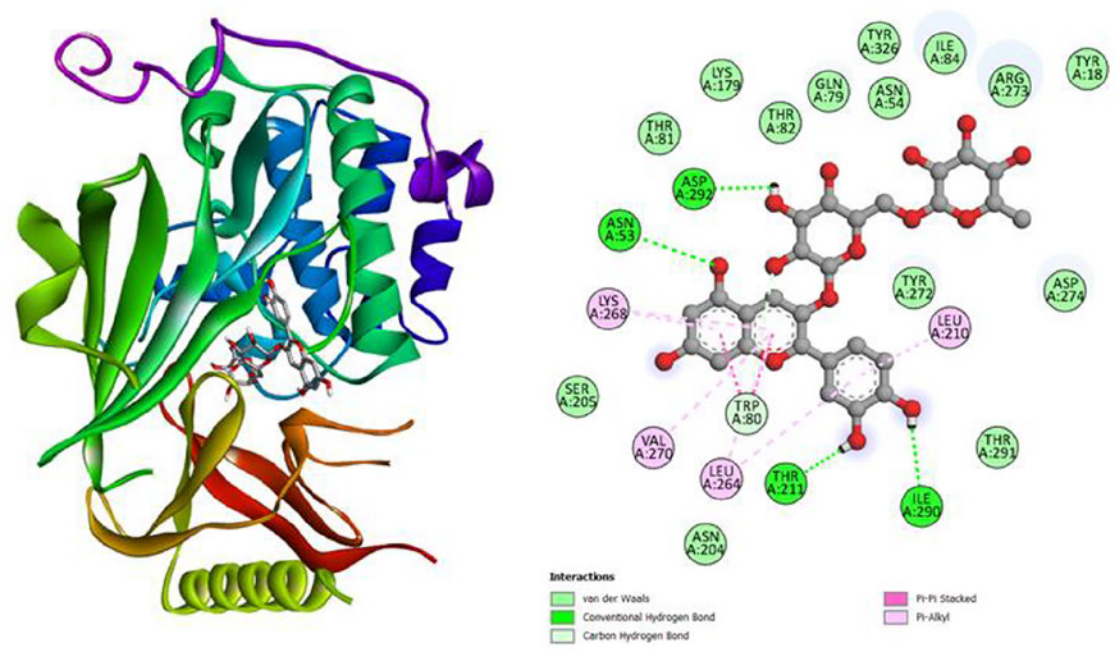

Keracyanin

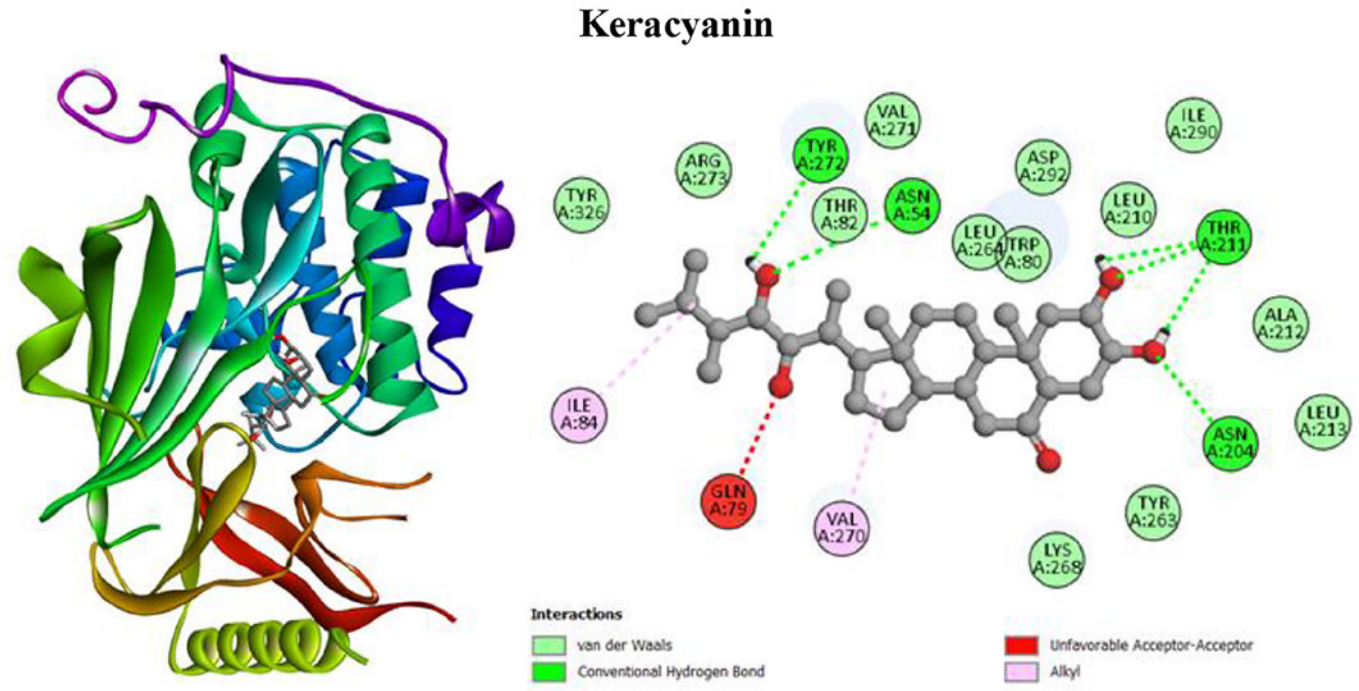

Castasterone

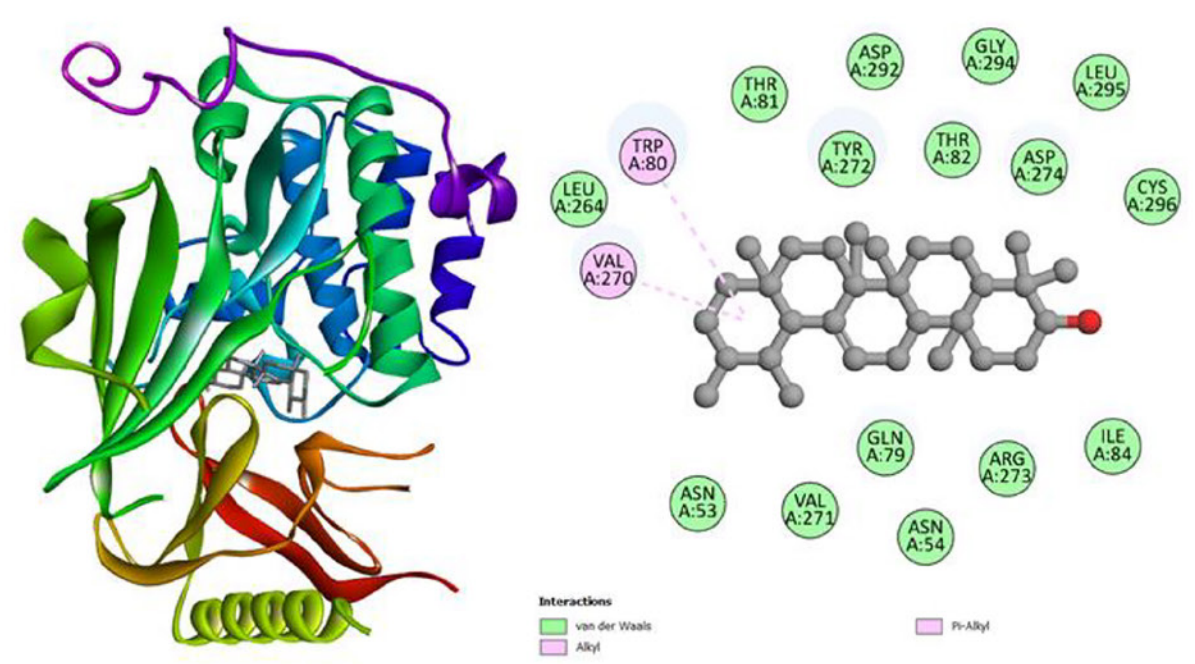

Alpha-amyrine

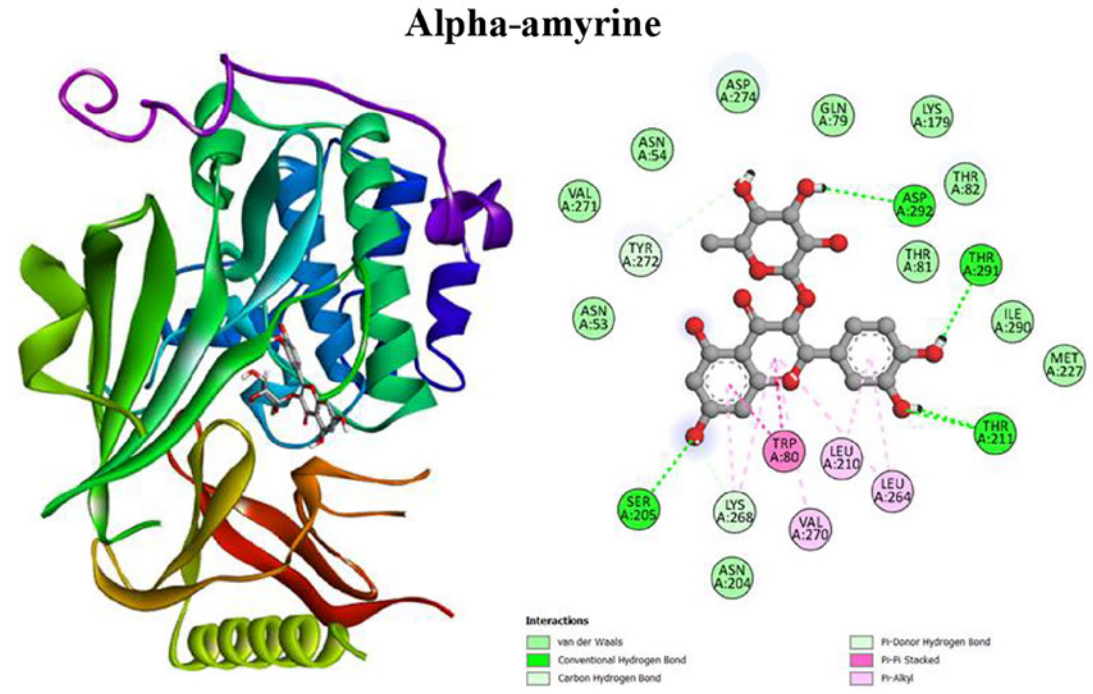

Quercetin

Supplementary Figure 1: Visualization of the molecular interactions between AKT1 with four buckwheat-derived phytochemicals.

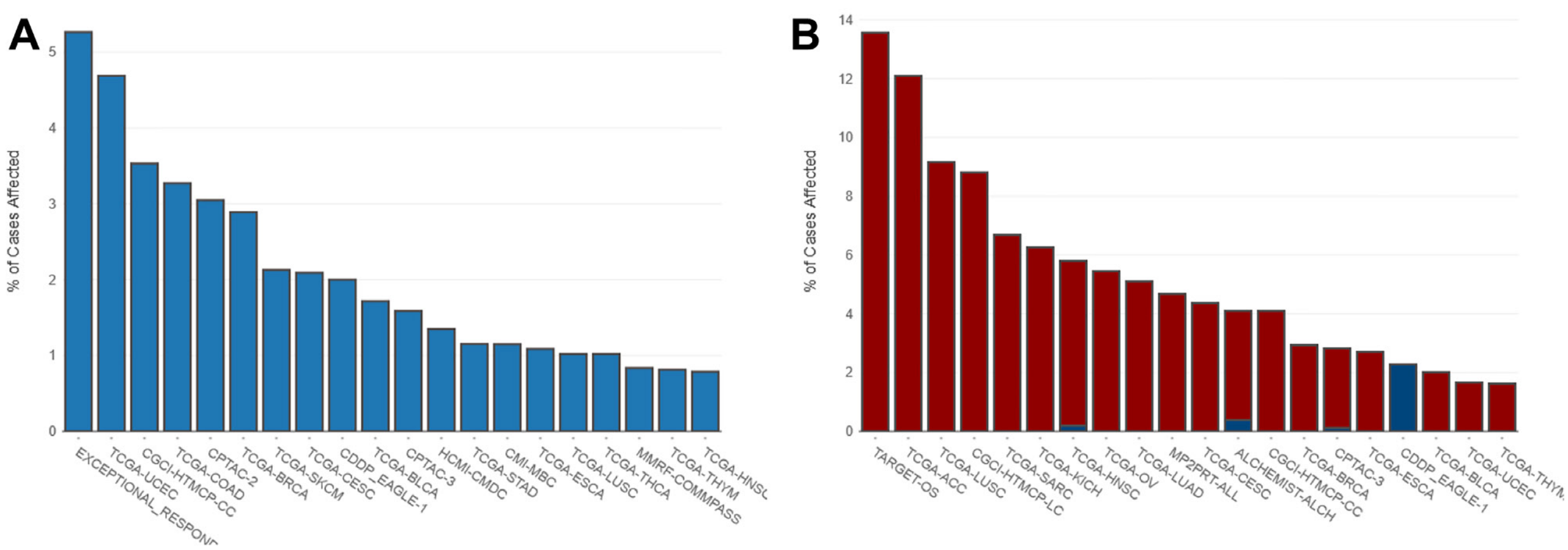

Supplementary Figure 2: Mutations (**A**) and CNV (**B**) of AKT1 identified from TCGA database.

Supplementary Table 1: Detailed molecular interactions of the top-ranked AKT1-ligand complexes

Compound	Binding Affinity (kcal/mol)	Residue	Distance (Å)	Interaction category	Interaction type
UC8	−14.40	CYS310	3.58	Hydrogen bond	Conventional H-bond
		TYR315	2.36	Hydrogen bond	Conventional H-bond
		TYR272	3.23	Hydrogen bond	Carbon H-bond
		ARG273	3.22	Electrostatic	π-Cation
		CYS296	4.07–4.19	Hydrogen bond	π-Donor H-bond / π-Sulfur
		VAL270	3.97	Hydrophobic	π-Sigma
		CYS310	4.96	Other	π-Sulfur
		TYR18	4.83	Hydrophobic	π-π Stacked
		TRP80	5.06–5.09	Hydrophobic	π-π Stacked
		TYR272	5.45	Hydrophobic	π-π Stacked
		TYR18	5.81	Hydrophobic	π-π Stacked
		LEU210	4.37	Hydrophobic	π-Alkyl
		LEU264	4.83	Hydrophobic	π-Alkyl
**Fagopyrine**	**−15.45**	VAL271	2.18	Hydrogen bond	Conventional H-bond
		LEU78	3.02	Hydrogen bond	Conventional H-bond
		ASN53	2.93	Hydrogen bond	π-Donor
		LEU264	3.48	Hydrophobic	π-Sigma
		VAL270	3.54	Hydrophobic	π-Sigma
		TRP80	3.78–5.53	Hydrophobic	π-π Stacked
		GLN79	4.62–4.73	Hydrophobic	Amide-π
		LYS268	4.07–5.03	Hydrophobic	π-Alkyl
		LEU210	5.20	Hydrophobic	π-Alkyl
		LEU264	5.25	Hydrophobic	π-Alkyl
		VAL270	4.17–5.09	Hydrophobic	π-Alkyl
**Keracyanin**	−11.94	ASN53	2.78	Hydrogen bond	Conventional H-bond
		ASP292	2.14	Hydrogen bond	Conventional H-bond
		ILE290	2.41	Hydrogen bond	Conventional H-bond
		THR211	2.26	Hydrogen bond	Conventional H-bond
		TRP80	3.27	Hydrogen bond	Conventional H-bond
		TRP80	3.92–4.62	Hydrophobic	π-π Stacked
		VAL270	4.90	Hydrophobic	π-Alkyl
		LYS268	4.17–5.35	Hydrophobic	π-Alkyl
		LEU210	4.21	Hydrophobic	π-Alkyl
		LEU264	5.11–5.28	Hydrophobic	π-Alkyl
**Castasterone**	−11.87	ASN54	2.95	Hydrogen bond	Conventional H-bond
		ASN204	2.53	Hydrogen bond	Conventional H-bond
		TYR272	2.91	Hydrogen bond	Conventional H-bond
		THR211	2.27–2.79	Hydrogen bond	Conventional H-bond
		VAL270	4.64	Hydrophobic	Alkyl
		ILE84	4.30	Hydrophobic	Alkyl

**α-Amyrin**	−11.62	VAL270	3.79	Hydrophobic	Alkyl
		TRP80	4.95	Hydrophobic	π-Alkyl
**Quercetin**	−11.21	SER205	2.66–3.66	Hydrogen bond	Conventional H-bond
		THR211	2.80–3.09	Hydrogen bond	Conventional H-bond
		THR291	2.85	Hydrogen bond	Conventional H-bond
		ASP292	2.25	Hydrogen bond	Conventional H-bond
		LYS268	3.28	Hydrogen bond	Conventional H-bond
		TYR272	3.25	Hydrogen bond	Conventional H-bond
		TRP80	3.74–4.40	Hydrophobic	π-π Stacked
		VAL270	5.24	Hydrophobic	π-Alkyl
		LYS268	4.12–5.27	Hydrophobic	π-Alkyl
		LEU210	4.22	Hydrophobic	π-Alkyl
		LEU264	5.06–5.32	Hydrophobic	π-Alkyl

Supplementary Table 2: Binding affinities of buckwheat-derived phytochemicals with AKT1

Phytochemical names	Binding affinity (Kcal/mol)
24-epi-Brassinolide	−11.1
beta-Amyrin	−10.9
Isoorientin	−10.74
Brassinolide	−10.68
Flavonol 3-O-D-glucoside	−10.44
Aromadendrin-3-o-beta-galactoside	−10.39
Orientin	−10.31
(-)-Epicatechin gallate	−10.25
Swertiamacroside	−10.12
Vitexin	−10.11
Hyperoside	−10.06
Isovitexin	−10.04
Isoquercitrin	−10.03
Kaempferol	−9.939
Quercetin	−9.891
3,8’-Biapigenin	−9.845
Procyanidin	−9.833
(-)-Epicatechin	−9.769
fagopyritol B3	−9.669
Cyanidin	−9.502
Cianidanol	−9.423
fagopyritol A3	−9.414
fagopyritol A2	−9.025
Squalene	−8.845
fagopyritol B2	−8.565
3,4-Dihydroxy-alpha-(2-piperidyl) benzyl alcohol hydrobromide	−7.884

fagopyritol A1	−7.72
fagopyritol B1	−7.537
Arachidonic acid	−7.448
Pyrosaccharopine	−7.088
Thiamine	−7.051
Linoleic acid	−6.908
Docosanoic acid	−6.897
Arachidic acid	−6.823
Oleic acid	−6.694
Stearic acid	−6.328
Vanillic acid	−6.244
3,4-Dihydroxybenzaldehyde	−6.148
Palmitic acid	−6.129
Phytic acid	−6.115
Methyl palmitate	−6.105
4-Hydroxyacetophenone	−6.073
Salicylaldehyde	−5.877
Ascorbic acid	−5.777
Fagomine	−5.455
